# Selective BRAFV600E Inhibitor PLX4720, Requires TRAIL Assistance to Overcome Oncogenic PIK3CA Resistance

**DOI:** 10.1371/journal.pone.0021632

**Published:** 2011-06-27

**Authors:** Eftychia Oikonomou, Michal Koc, Vladimira Sourkova, Ladislav Andera, Alexander Pintzas

**Affiliations:** 1 Laboratory of Signal Mediated Gene Expression, Institute of Biological Research and Biotechnology, National Hellenic Research Foundation, Athens, Greece; 2 Laboratory of Cell Signaling and Apoptosis, Institute of Molecular Genetics, Czech Academy of Sciences, Prague, Czech Republic; Karolinska Institutet, Sweden

## Abstract

Documented sensitivity of melanoma cells to PLX4720, a selective BRAFV600E inhibitor, is based on the presence of mutant BRAF^V600E^ alone, while wt-BRAF or mutated KRAS result in cell proliferation. In colon cancer appearance of oncogenic alterations is complex , since BRAF, like KRAS mutations, tend to co-exist with those in PIK3CA and mutated PI3K has been shown to interfere with the successful application of MEK inhibitors. When PLX4720 was used to treat colon tumours, results were not encouraging and herein we attempt to understand the cause of this recorded resistance and discover rational therapeutic combinations to resensitize oncogene driven tumours to apoptosis. Treatment of two genetically different BRAF^V600E^ mutant colon cancer cell lines with PLX4720 conferred complete resistance to cell death. Even though p-MAPK/ ERK kinase (MEK) suppression was achieved, TRAIL, an apoptosis inducing agent, was used synergistically in order to achieve cell death by apoptosis in RKO*^BRAFV600E/PIK3CAH1047^* cells. In contrast, for the same level of apoptosis in HT29*^BRAFV600E/PIK3CAP449T^* cells, TRAIL was combined with 17-AAG, an Hsp90 inhibitor. For cells where PLX4720 was completely ineffective, 17-AAG was alternatively used to target mutant BRAF^V600E^. TRAIL dependence on the constitutive activation of BRAF^V600E^ is emphasised through the overexpression of BRAF^V600E^ in the permissive genetic background of colon adenocarcinoma Caco-2 cells. Pharmacological suppression of the PI3K pathway further enhances the synergistic effect between TRAIL and PLX4720 in RKO cells, indicating the presence of PIK3CA^MT^ as the inhibitory factor. Another rational combination includes 17-AAG synergism with TRAIL in a BRAF^V600E^ mutant dependent manner to commit cells to apoptosis, through DR5 and the amplification of the apoptotic pathway. We have successfully utilised combinations of two chemically unrelated BRAF^V600E^ inhibitors in combination with TRAIL in a BRAF^V600E^ mutated background and provided insight for new anti-cancer strategies where the activated PI3KCA mutation oncogene should be suppressed.

## Introduction

For a long time it has been appreciated that presence of KRAS mutations highly correlates with colorectal cancer (CRC) progression and decreased patient survival. In addition, the more recently identified BRAF mutations in CRC, do not co-exist with those in KRAS and display a more potent transforming activity to be associated with progression to metastasis [1], [2]. Notable among the apoptosis-inducing stresses are signalling imbalances resulting from elevated levels of oncogene signalling, as mentioned earlier, and DNA damage associated with hyper-proliferation. In contrast to KRAS mutations, those in BRAF have the ability to cause genomic rearrangements in colon cells that can potentially sensitize them to apoptosis a major advantage in cancer therapeutics, since deregulation of apoptosis can lead to growth advantage in cancer cells. Yet other research has revealed how apoptosis is attenuated in those tumours that succeed in progressing to states of high-grade malignancy and resistance to therapy [3], [4]. The goal of most anti-tumour therapies, including chemotherapy, radiation or newer targeted therapies is to ultimately induce the death of tumour cells. Different chemotherapies induce death of tumour cells by different mechanisms. These include both apoptotic forms of cell death, as well as non-apoptotic mechanisms such as autophagy, necrosis and mitotic catastrophe [5]. However, the fraction of tumour cells that undergo non-apoptotic death are significantly increased if apoptosis-related mechanisms are inhibited [6]. Constitutive activation of MAPK has been found in many different tumor cell lines and primary tumors including colon cancer cells and tissues [7]–[9]. High expression and constitutive activation of PI3K is also found in gastric cancer and CRC [10]. Prominent among cell surface molecules capable of initiating and tightly controlling apoptosis in cancer cells is TRAIL, rendering it a promising anti-cancer agent [11], [12].

TRAIL induces apoptosis via interacting with its death receptors (DRs), DR4 and DR5, which in turn results in death-inducing signalling complex (DISC) formation and caspase-8 processing [13]. Caspase-8 activation can then result in caspase-3 activation through the mitochondrial-independent pathway, and/or through the activation of Bid, a pro-apoptotic BH3-only Bcl-2 family member, which when cleaved induces the mitochondrial release of apoptogenic factors such as Bax and Bak through the mitochondrial-dependent pathway [14]. Despite the fact that during colorectal carcinogenesis a marked increase in sensitivity to TRAIL has been reported, cells like HT29 and RKO remain partially resistant to TRAIL-induced apoptosis [15].

The MAPKs that are activated by phosphorylation may also act as important modulators of various apoptosis-inducing signals, while the protective effect of extracellular signal-related kinase 1/2 (ERK1/2) on DR-induced apoptosis has been described [16], [17]. Deregulation of this pathway by RAS and more recently BRAF oncoproteins induce constitutive ERK1/2 activation, thereby promoting cell growth and survival. Manipulation of the MAPK signalling pathway could be a powerful means of treatment for tumours with BRAF mutations especially those resistant to TRAIL. Difluorophenyl-sulfonamine (PLX4720) targeting BRAF^V600E^ cancer cell proliferation has recently been described as potent and selective among the many tested clinically [18]. However application and dosage of this inhibitor should be scrutinised since in can bind wild-type BRAF in KRAS mutant cells and activate the MAPK pathway through CRAF The mechanism of RAF regulation presents another strategy for its inhibition [19]–[21].

The protein chaperone Hsp90 is required for the conformational maturation of several key signalling proteins, including PI3K, p-Protein Kinase B (pAKT), NF-kB, CRAF and more recently BRAF^V600E^ [22], [23]. Inhibition of Hsp90 function with a geldanamycin derivative, 17-allylamino-17-demethoxygeldanamycin (17-AAG), has been shown to effectively inhibit Hsp90 function of malignant cells *in vivo* due to its great affinity for the activated polymerized form of the molecular chaperone at tolerable doses [24]. Although CRAF and ARAF are degraded in cells that are exposed to 17-AAG, wild-type BRAF is not found in an Hsp90 complex, while BRAF^V600E^ degradation leads to MAPK inhibition, cell-cycle arrest, and apoptosis with concomitant antitumour activity *in vivo* [22].

Here we compare the selective BRAF^V600E^ inhibitor PLX4720 with the Hsp90 inhibitor 17-AAG, in colon cancer cells. Recorded resistance to PLX4720 is attributed to the activating PIK3CA mutations that coexist with BRAF^V600E^. Treatment with 17-AAG is more responsive, potentially due to the multiple oncogenic proteins that are Hsp90 clients. Cell death by apoptosis at these conditions is significantly facilitated when TRAIL is concomitantly administered, as compared to the innate sensitivity to TRAIL in colon cancer cells bearing a single BRAF^V600E^ mutation.

## Materials and Methods

### Growth Inhibition Studies and Cytoxicity Assays

The Caco-2, Colo205, HT29, RKO, DLD-1 and SW620 human colon adenoma-carcinoma cell lines were obtained from ATCC. The Caco-BR clones constitutively expressing active BRAF^V600E^ proteins and the Caco-NEO9-empty vector clones have been previously described [1], [25]. For growth inhibition and cytoxicity studies we used the sulforhodamine B (SRB) assay. Methodology is listed in supplementary materials and methods (File S1). For assessment and quantification of TRAIL-induced apoptosis, the ELISA cell death kit by Roche (Indianapolis, IN) was alternatively used according to the manufacturer's protocol that is highly sensitive and detects apoptotic nucleosomes in cell lysates.

For blocking experiments cells were pre-incubated for 15 minutes with 2 µg/ml of the respective blocking antibody against DR4 and DR5 and then stimulated with TRAIL with and without pretreatment with 17-AAG. Photographs were taken using a Nikon Eclipse T-200 (Tokyo, Japan) inverted phase-contrast microscope equipped with an Olympus digital camera (Olympus SP-51OU2, Hamburg, Germany).

### Three-Dimensional Culture

For three-dimensional culture experiments, cells were grown in 24-well plates on 20% Matrigel that was allowed to set for 15 minutes at 37°C in order to form a gel of 1 mm thickness. The bottom later was then covered with 2×10^4^ cells mixed 1∶1 with 4% Matrigel in a total volume of 600 µl. Growth medium containing 2% matrigel was replaced every 2 days and the cells were left to grow for 12–14 days to allow development of extensive tubule network, after which treatment were applied for indicated incubation times. Photographs of the three-dimensional cultures were taken using a Nikon Eclipse T-200 inverted phase-contrast microscope equipped with an Olympus digital camera. The nuclei were stained with Hoechst No. 33342.

### Immunoblotting and Immunoprecipitation

Whole cell lysates were prepared with Nonidet P-40 (NP-40) buffer containing protease inhibitors and were subject to Western blot analysis or immunoprecipitation studies. Methodology and antibody information is listed in supplementary materials and methods (File S1). For immunoprecipitation of the NP-40 insoluble fractions, pellets were resuspended in NP-40 lysis buffer and sonicated three times for 10 sec at 4°C using an MSE Soniprep150 to give the insoluble fraction.

### Suppression of BRAF^V600E^ and PIK3CA^H1047^ expression by RNA interference

The small inhibitory duplex shRNA oligo was cloned into the *Hind*III and *Bgl*II sites in pSUPER (Oligoengine, Seattle, WA). The sense strand of the shRNA pSUPER BRAF^V600E^ insert was BRAFmutshRNA: gatccccGCTACAGAGAAATCTCGATttcaagagaATCGAG-ATTTCTCTGTAGCtttttggaaa (Hingorani et al., 2003). BRAFmutshRNA or vector control (pSUPER) plasmids or siRNA- PIK3CA^H1047^ (Darmacon) were transiently expressed into cells using lipofectamin (Sigma).

### DISC Analysis

The ligand affinity precipitation was done using biotin-conjugated TRAIL (Bio-TRAIL) in combination with streptavidin-agarose beads. Methodology and antibody information is listed in supplementary materials and methods (File S1). Ligand affinity precipitates were washed 5 times with lysis buffer and the protein complexes were eluted from the beads by the addition of 30 µl SDS sample buffer and heating at 95°C for 15 minutes. Proteins were separated in SDS-PAGE and immunoblotted.

### Immunofluorescence Microscopy

Immunostaining methodology is listed in supplementary materials and methods (File S1). Briefly, cells were incubated with indicated primary antibodies for 2 hours at room temperature, while the secondary antibody was applied to the cells for 1 hour also at room temperature. The nuclei were stained with Hoechst and visualized with a Leica TCS SPE confocal laser scanning microscope (Leica Lasertechnik, Heidelberg, Germany).

### Flow cytometry and apoptosis assays

For immunostaining, cells were incubated with 50 µg/ml anti-DR4 or anti-DR5 on ice for 30 minutes. Surface expression of the receptors on living cells (Hoechst negative) was analyzed using a LSRII flow cytometer (BD Biosciences). M30 Cytodeath assay of caspase-3-cleaved cytokeratine 18 or Annexin V-FITC/ Hoechst staining were used for the assessment and quantification of TRAIL-induced apoptosis according to the manufacturer's protocols. Methodology is listed in supplementary materials and methods (File S1).

### Semiquantitative RT-PCR analysis

Total RNA was extracted using TRIzol reagent (Invitrogen, Karlsruhe, Germany). The extracted total RNA (3 µg) was reverse transcribed into cDNA using the SuperScriptt II Reverse Transcriptase (Invitrogen) according to the manufacture. RT–PCR amplification was performed as previously described and intensity values were measured using Molecular Dynamics ImageQuant Software. All PCR products were normalized to GAPDH expression [26].

## Results

### A small therapeutic window for PLX4720 efficacy in BRAF mutant cells

**Figure 1:**
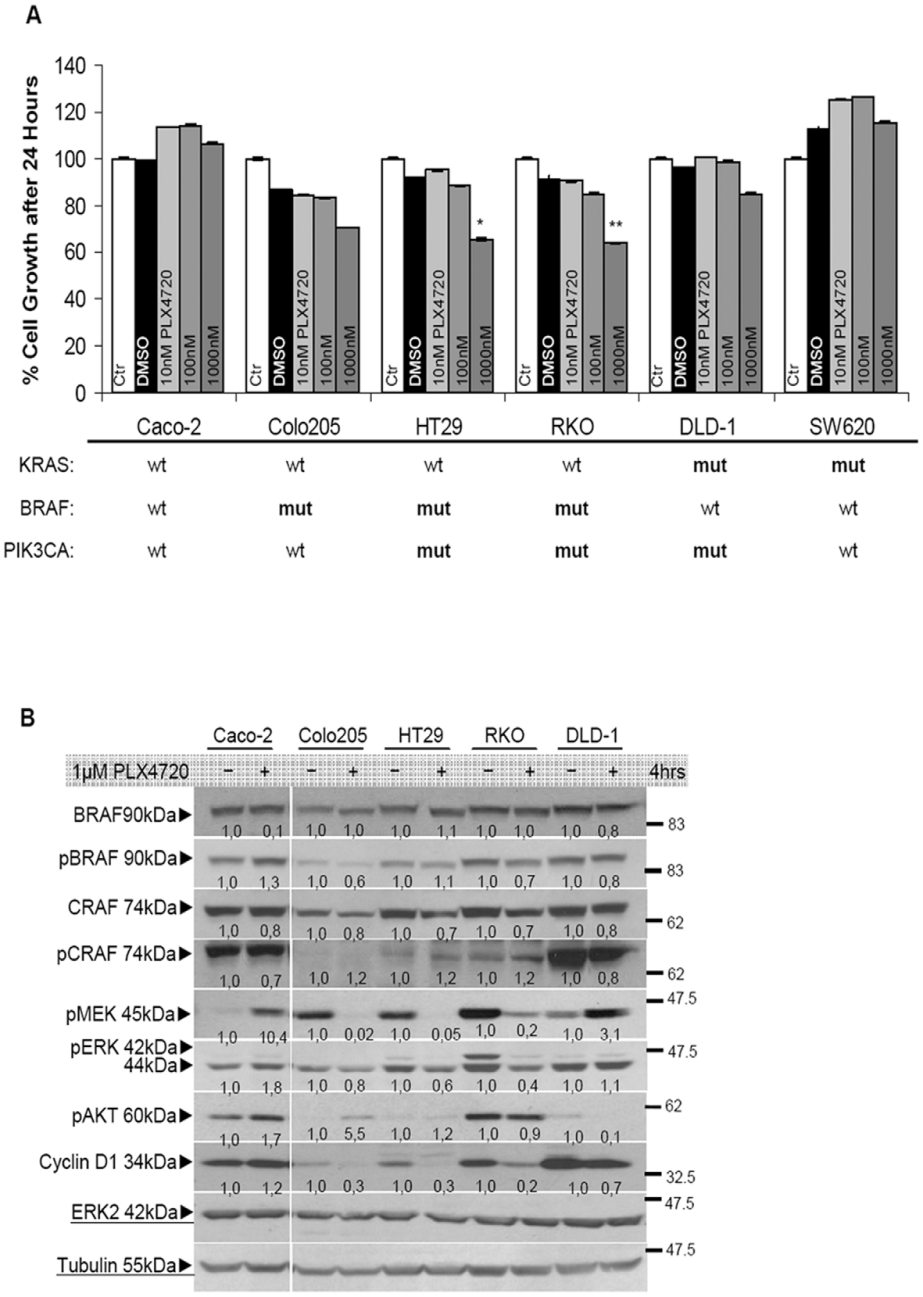
Presence of BRAF mutations are not selective for efficient PLX4720 treatment in colon cancer cells. (**A**) Cell survival for a panel of colon cancer cell lines treated with the selective for mutant BRAF pharmacologic inhibitor PLX4720. The values are the average of three independent experiments and are presented as fold change of the absorbance of treated/ untreated cells, for each condition. The number of viable cells was assayed by SRB 24 hours after treatment. Treatment conditions are provided along the top on each column. *P<0.05, **P<0.01 vs Ctr-untreated. (**B**) Total cell lysates harvested from cells treated for 4 hours with 1 µM PLX4720 were immunoblotted for the indicated antibodies. Proteins are quantified against α-tubulin and pERK levels against total ERK using the untreated condition of each cell line as a reference to the treated one.

To identify molecular modifiers in currently resistant cells that influence the sensitivity of BRAF mutant cells to TRAIL induced apoptosis, we recorded a panel of colon cancer cell lines to determine their response to the BRAF inhibitor PLX4720, which targets the mutated V600E form of BRAF. Given the frequent co-occurrence of KRAS/ BRAF and PI3K pathway lesions, two of the selected cell lines harbored both BRAF and PI3K pathway mutations. In response to PLX4720 no concentration dependence was observed for any of the cell lines tested and only a small inhibitory effect was achieved upon treatment with the highest concentration in HT29 and RKO cells (Figure 1A). Treatment with PLX4720 managed to inhibit the phosphorylation of BRAF kinase to a degree as compared to the increased pCRAF levels only in the BRAF^V600E^ mutant cell lines (Colo205, RKO and HT29). Further downstream the MAPK pathway, the inhibitory effect was more pronounced with a sustained inhibition mainly on pMEK and pERK levels in cell lines harboring BRAF and PI3K pathway mutations. Sustained inhibitory effect on the MAPK pathway had an immediate effect on cyclin D1 target gene, all be it in a much shorter (1–4 hours) incubation period (Figure 1B, Figure S1). The inhibitory effect of PLX4720 on MEK pathway in shorter rather than longer incubation periods indicates a potentially small therapeutic window for treatment efficacy. As expected, cell lines that are wild-type for BRAF (Caco-2) or KRAS mutant (DLD-1) increased their proliferation rate by about 20% as compared to control untreated cell following treatment with PLX4720, independently of their doubling, that was also described by the increase of pMEK and pERK levels in these cells (Figure 1A–B).

### Potent inhibition of cell growth in BRAF^MT^ and activated BRAF^WT^ cells using 17-AAG

**Figure 2:**
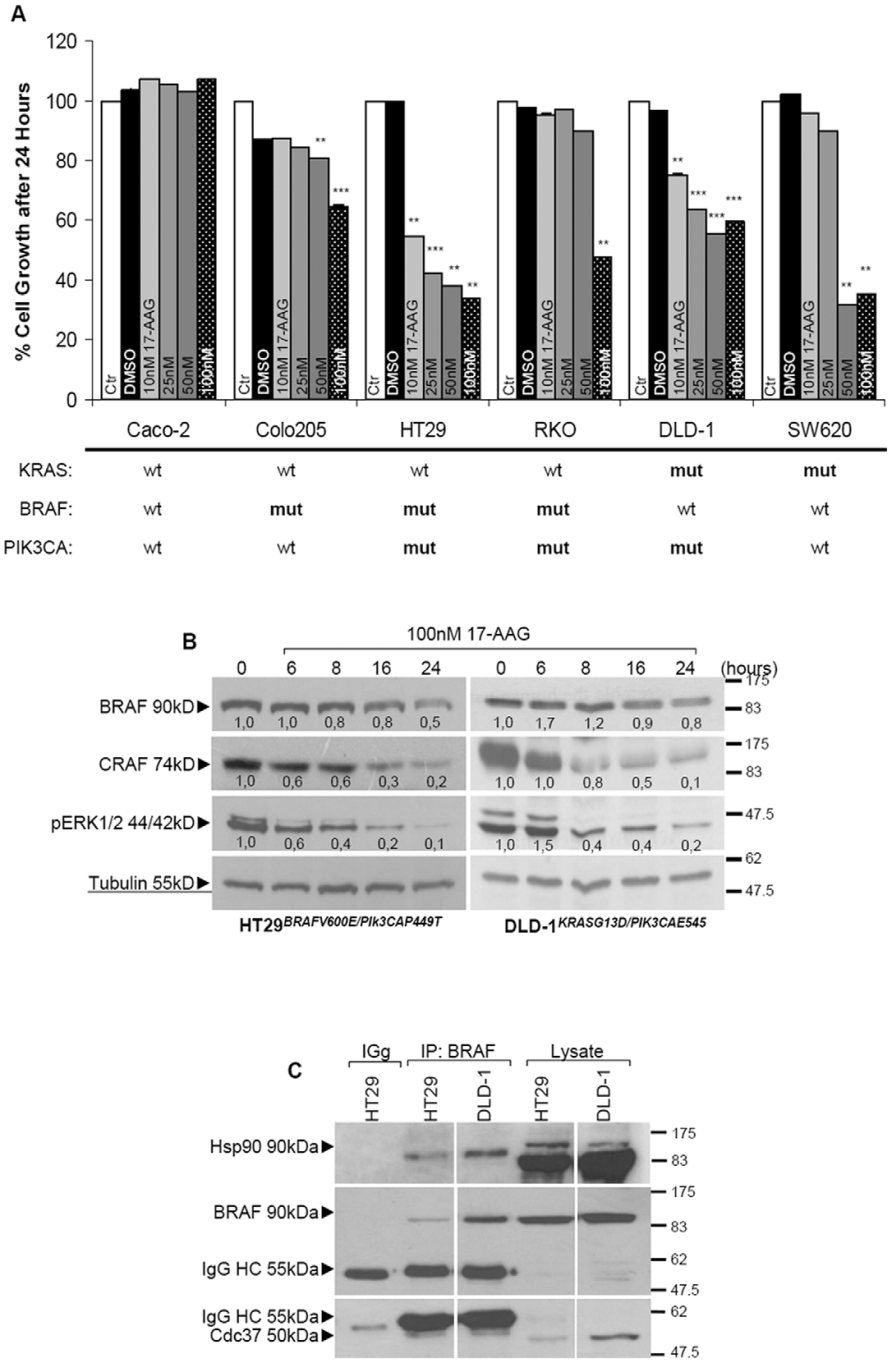
Presence of BRAF^V600E^, an Hsp90 client protein, confers sensitivity to 17-AAG in colon cancer cells. (**A**) Cell survival for a panel of colon cancer cell lines treated with the Hsp90 pharmacologic inhibitor 17-AAG. The values are the average of three independent experiments and are presented as fold change of the absorbance of treated/ untreated cells, for each condition. The number of viable cells was assayed by SRB 24 hours after treatment. Treatment conditions are provided along the top on each column. *P<0.05, **P<0.01, ***P<0.001 vs Ctr-untreated. (**B**) Total cell lysates harvested from cells treated for indicated time points with 100 nM 17-AAG were immunoblotted for the indicated antibodies. Proteins are quantified against α-tubulin. (**C**) Total cell lysates from indicated cell lines were immunoprecipitated with BRAF and the complexes were immunoblotted with Hsp90, BRAF and Cdc37, which is right bellow the heavy chain (HC) of the antibody.

Attenuation of the MAPK pathway through mutant BRAF inactivation by 17-AAG, which inhibits Hsp90 function, could underline reversal of observed TRAIL resistance. The same panel of colon cancer cell lines was monitored for their response to 17-AAG inhibitor. Significant cytotoxicity was observed in one BRAF mutant cell line (HT29), while considerable was the extent of cell toxicity in Colo205, RKO and SW620, cell lines with distinct genetic background. Nevertheless, sensitivity seemed to correlate to pATK levels rather than the PI3K mutation itself (Figure 2A, 1B). Regardless the co-occurrence of BRAF and PI3K pathway mutations in RKO and HT29 cells, basal pAKT levels were significantly increased only in the RKO cells indicating a diminished activating potential for the PIK3CA mutation (P449T) present in HT29 cells. Notably, sensitivity to 17-AAG was observed only in cell lines with moderate pAKT levels (HT29, DLD-1, Colo205 and SW620) (Figure 2A, 1B). Degradation of mutant BRAF in HT29 cells but also wild-type BRAF in DLD-1 cells, activated by co-expression with mutant KRAS, account for the inhibitory effect observed in cell growth (Figure 2B). To determine whether BRAF^V600E^ is an Hsp90 client protein, we tested its sensitivity using as a control CRAF, a known Hsp90 client. CRAF expression was much more rapidly and efficiently degraded. As expected, the reduction in CRAF was accompanied by decreased ERK phosphorylation only in BRAF/ KARS mutant cells (Figure 2B). By testing whether BRAF and Hsp90 associate with each other, we established that both mutant BRAF and wild-type BRAF activated by the upstream mutant KRAS, are Hsp90 client proteins in HT29 and DLD-1 cells respectively. The kinase-specific Hsp90 co-chaperone p50cdc37 was also found to co-precipitate with mutant BRAF (Figure 2C, Figure S2). Surprisingly, as previously shown by Dias and colleagues, we also did not observe any binding of CRAF to Hsp90 in any cell line (data not shown) possibly for reasons previously discussed [27]. Taken together, either mutated or activated BRAF is more dependent on Hsp90-chaperone for its folding, stability and oncogenic activity than wild-type inactive protein.

### PLX4720 cooperates with TRAIL to commit cells to apoptosis

**Figure 3:**
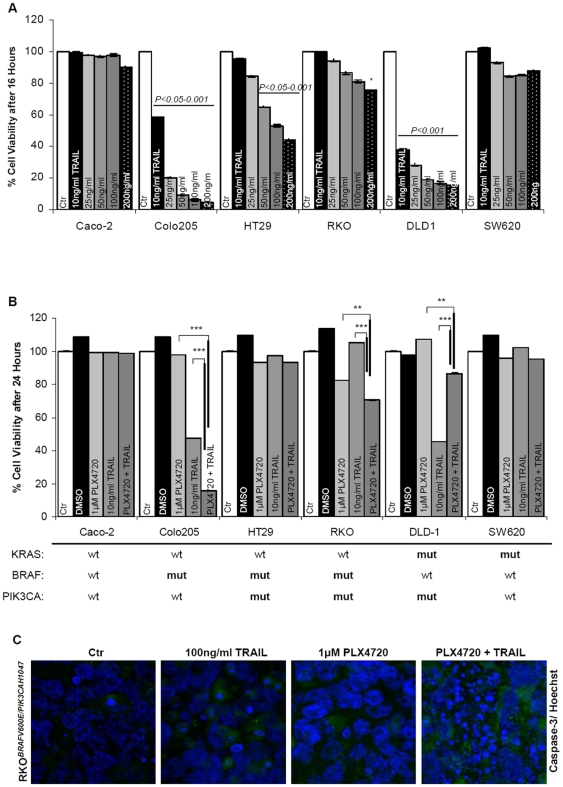
PLX4720 sensitises RKO cells to TRAIL-induced apoptosis. (**A**) Cytotoxic effects of the apoptotic agent TRAIL in a panel of colon cancer cell lines. The values are the average of three independent experiments and are presented as fold change of the absorbance of treated/ untreated cells, for each condition. The number of viable cells was assayed by SRB 16 hours after treatment. Treatment conditions are provided along the top on each column. *P<0.05, **P<0.01, ***P<0.001. (**B**) Combined treatment with 1 µM PLX4720 for 8 hours and concomitant administration of 10 ng/ml TRAIL for 16 hours in a panel of colon cancer cell lines. Cell viability assayed by SRB 24 hours after treatment. (**C**) Caspase-3 activation analysed by immunofluorescence 24 hours after induction with TRAIL/ PLX4720 alone or in combination, in a 3D culture of RKO cells. Representative confocal immunofluorescence images, double labelled with Hoechst (blue) and active caspase-3 (green). Original magnification 63x.

In an attempt to render resistant colon cancer cell more sensitive to RAF inhibitor treatment with PLX4720, TRAIL was used as a synergistic agent. Resistance to TRAIL treatment as a single agent appeared to strongly correlate with the PI3K pathway mutational status. TRAIL conferred limited cytotoxicity in BRAF mutant cells harboring activating mutations in PIK3CA whereas, extensive apoptosis was recorded in cells like Colo205 harboring a single BRAF mutation, at very small doses (10 ng/ml), comparable to the sensitive DLD-1 cells (Figure 3A). As expected, highly metastatic SW620 colon carcinoma cells were completely resistant to TRAIL as were also the intermediate adenocarcinoma Caco-2 cells. Treatment of cells with PLX4720 followed by co-concomitant administration with the lowest dose of TRAIL (10 ng/ml) induced increased cell death only in cells (RKO) harboring a BRAF mutation and high pAKT levels. The combination of PLX4720 with TRAIL was not beneficial to already sensitive to TRAIL cells, but was additive in cells harboring a BRAF mutation only (Colo205) (Figure 3B). Notably previously sensitive to TRAIL DLD-1 cells were rescued during the combined treatment of TRAIL with PLX4720 suggesting that the presence of wild-type BRAF may interfere with the apoptotic outcome. Conferred resistance to the therapeutic combination by wild-type BRAF was also confirmed in two independent and genetically similar colon cancer cell lines, DLD-1 and HCT116 (Figure S3), indicating only wild-type BRAF as the exclusionary factor for colon cancer treatment with PLX4720.

The combination efficacy of BRAF inhibition and TRAIL treatment was also applied to a three-dimensional (3D) cell culture system as an *in vitro* tumor model where a significant apoptotic effect was recorded. After two weeks of cell growth and tubule formation within the spheroid structures, PLX4720/ TRAIL induced caspase-3 activation and nuclear fragmentation was evident (Figure 3C). This provides further evidence that this combinatorial treatment can also be efficient *in vivo.*

### Combined inhibition of BRAF and PIK3CA increases TRAIL induced apoptosis in RKO cells

**Figure 4:**
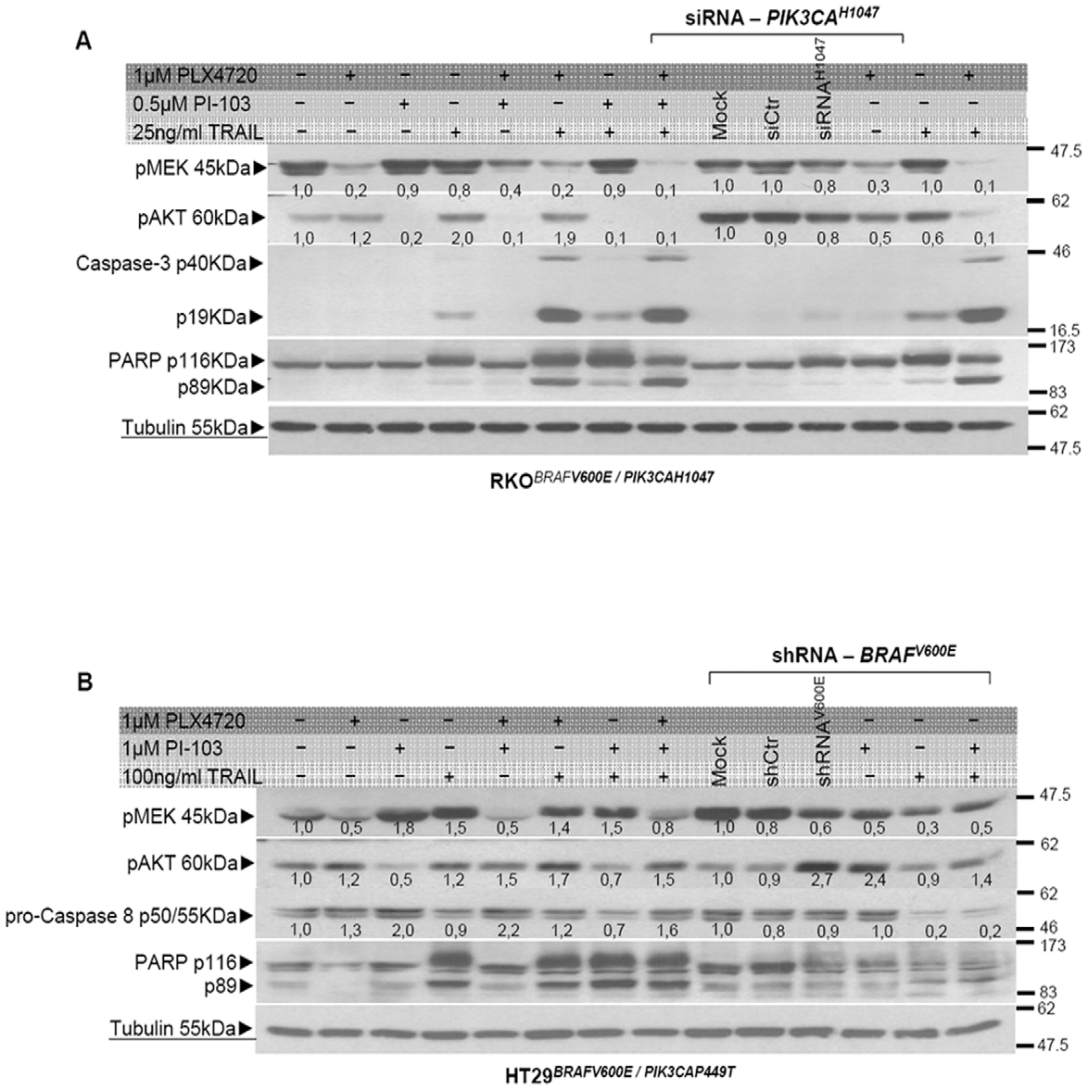
Inhibition of PIK3CA increases the combination efficacy of TRAIL with PLX4720 in RKO cells. (**A**) Cells left untreated or treated with either 1 µM PLX4720 or a PIK3CA pharmacologic inhibitor PI-103 (0.5 µM) for 24 hours. Combined treatment of PLX4720 for 8 hours with concomitant administration of 25 ng/ml TRAIL for 16 hours was performed along side with TRAIL treatment alone for 16 hours. Combined inhibition of mutant PI3K and BRAF using 0.5 µM PI-103 and 1 µM PLX4720 for 8 hours following concomitant administration of 25 ng/ml TRAIL for 16 hours. Alternative depletion of mutant PIK3CA by transient transfection of 1.6 pmol siRNA against the PIK3CAH1047 present in RKO cells in combination with all indicated parameters. Total cell lysates harvested from the indicated treatments were analyzed for the phosphorylation of downstream targets and induction of apoptotic markers. (**B**) Cells left untreated or treated with either 1 µM PLX4720 or 1 µM PI-103 for 24 hours. Combined treatment with PLX4720 for 8 hours and concomitant administration of 100 ng/ml TRAIL for 16 hours was performed along side with TRAIL treatment alone for 16 hours. Combined inhibition of mutant PI3K and BRAF using PI-103 and PLX4720 following concomitant administration of TRAIL and alternative depletion of mutant BRAF by transient transfection of 7 µg shRNA against the BRAFV600E present in HT29 cells in combination with all indicated parameters.

The antiproliferative effect in response to the combined treatment involving the mutant BRAF inhibition and the induction of the apoptotic TRAIL pathway could be related to the PI3K mutational status. In order to answer this question, the molecular mechanism underlying this effect, was analyzed. Depletion of either mutant BRAF or PIK3CA, using PLX4720 or the PI-103 inhibitor, that block PI3K signaling downstream of p110a, did not induce any apoptotic response (Figure 4A, lanes 2–3) nor did their combination (Figure 4A, lane 5). When TRAIL was used alone, partial apoptosis was recorded (Figure 4A, lane 4). However, combining the inhibition of mutant BRAF with TRAIL treatment induced several markers of apoptosis including cleavage of Poly ADP (Adenosine Diphosphate)-Ribose Polymerase (PARP) and caspases-3 activation (Figure 4A, lane 6). Inhibition of PIK3CA and concomitant TRAIL treatment did not induce a significant apoptotic response (Figure 4A, lane 7). A more potent growth-inhibitory effect was elicited by the combination of the PI3KCA inhibition and the concomitant treatment with PLX4720 and TRAIL in RKO cells that resulted in increased apoptotic cell death (Figure 4A, lane 8). Replacement of the PIK3CA-p110a inhibitor (PI-103) with a siRNA against the PIK3CA^H1047^ mutant in RKO, did not manage to increase apoptotic cell death any further as compared to PI-103 (Figure 4A, lanes 13–14). BRAF depletion using shRNA against mutant BRAF instead of PLX4720 likewise proved less efficient in co-treatment protocols involving TRAIL (Figure S4).

### Inhibition of PIK3CA increases TRAIL induced apoptosis in HT29 cells

When the same experimental approach was applied to HT29 cells, the combination of BRAF inhibition using PLX4720 and TRAIL treatment was not any more advantageous than TRAIL alone (Figure 4B, lanes 4 and 6). Depletion of PIK3CA using PI-103 and concomitant treatment with TRAIL, rendered cells slightly more prone to apoptosis, evident by increased PARP cleavage and the decrease in pro-caspase-8 protein expression (Figure 4B, lane 7). Nevertheless, the combination of the PI3KCA inhibitor and concomitant treatment with PLX4720 and TRAIL did not increase apoptotic cell death in HT29 cells that are genetically similar to the RKO cell line and sensitive to the combination just described (Figure 4B, lane 7). Even though, the shRNA against mutant BRAF in RKO cells was not very efficient, possible because of highly activated BRAF, its application in HT29 seems to be able to sequester pMEK about 2-fold (Figure 4B, lane 11) without any obvious impact in amplifying apoptosis for any of attempted combination treatments.

### 17-AAG overcomes TRAIL resistance in HT29 cells through enhanced apoptosis

**Figure 5:**
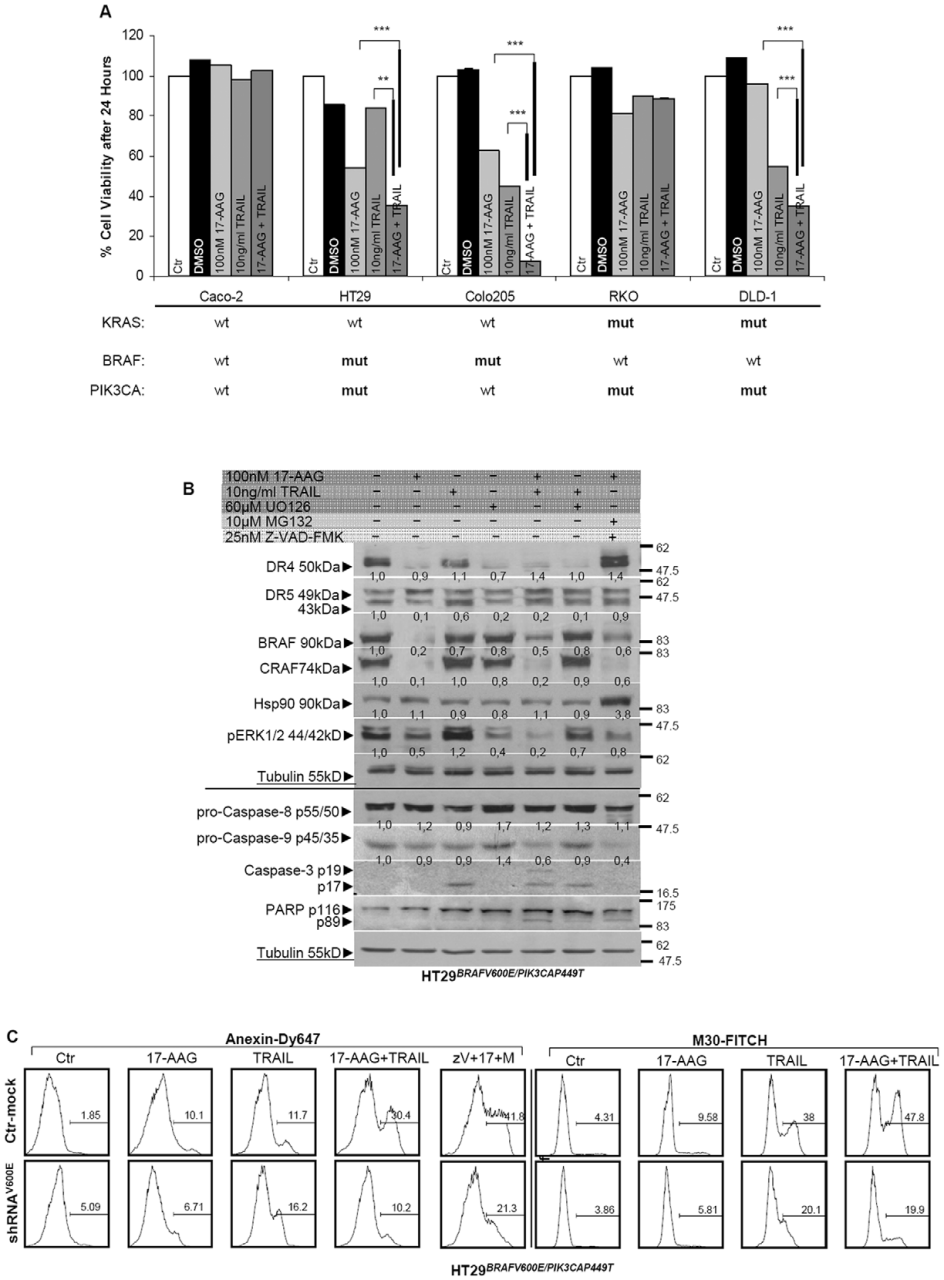
17-AAG sensitises HT29 cells to TRAIL-induced apoptosis. (**A**) Cytotoxic effects of the combined treatment using 17-AAG and concomitant administration of TRAIL in a panel of colon cancer cell lines. The values are the average of three independent experiments and are presented as fold change of the absorbance of treated/ untreated cells, for each condition. The number of viable cells was assayed by SRB 24 hours after treatment. Treatment conditions are provided along the top on each column. *P<0.05, **P<0.01, ***P<0.001 vs Ctr-untreated. (**B**) Cells left untreated or treated with 100nM 17AAG for 24 hours. Combined treatment of 17-AAG for 8 hours and concomitant administration of 100 ng/ml TRAIL for 16 hours was performed along side with TRAIL treatment alone for 16 hours. Alternatively cells were pre-treated with 25 nM Z-VAD-FMK for 1 hour then 10 µM MG132 was added for another hour after which cell were treated with 100 nM 17-AAG for 22 hours. Finally cells were pre-treated pretreated with 60 µM UO126 for 8 hours after which TRAIL was added for another 16 hours. (**C**) Depletion of mutant BRAF by transient transfection of 7 µg shRNA against the BRAF^V600E^ present in HT29 cells in combination with all indicated parameters described above. Apoptotic cell death after 24 hours was assayed using the Annexin V and M30 CytoDEATH assay.

Pretreatment of colon cancer cells with 17-AAG following treatment with TRAIL significantly sensitized HT29 to TRAIL and had a more mild effect on DLD-1 cells. As previously, the combination of 17-AAG with TRAIL was not beneficial to cells already sensitive to TRAIL (Colo205), nor seemed to be related to the mutational background of the cells (Figure 5A). The synergistic effect of 17-AAG and TRAIL significantly increased cell death as compared to each drug alone, while the sensitivity to 17-AAG did not correlate with the combination's cytotoxicity.

Sensitivity to pretreatment with 17-AAG appeared to be directly related to BRAF^V600E^ protein degradation in HT29 cells (Figure 5B-upper panel). TRAIL alone has a sub-toxic effect on partially resistant HT29 cells, while their combinatorial treatment resulted in procaspase-8 processing and Bid cleavage leading to caspase-3 activation and PARP cleavage. This suggests amplification of the mitochondrial pathway to mediate apoptosis (Figure 5B-lower panel). Independent caspase-9 processing in the presence of 17-AAG alone, suggests a mechanism of activation dependent on degradation of either BRAF^V600E^ or Hsp90 itself (Figure 5B). To determine whether this gained sensitivity to apoptosis was dependent on the mutant BRAF and not a non-specific effect of 17-AAG on other Hsp90 client proteins, attenuation of mutant BRAF in HT29 cells by shRNA^V600E^ managed to decrease apoptotic cell death by 17-AAG (Figure 5C). Evidence of significantly impaired apoptosis was evaluated using the M30 CytoDEATH assay, which measures a cleaved fragment of cytokeratin 18 and Annexin V that measures apoptotic fractions (Figure 5C). Interaction between Hsp90 and mutant BRAF was impeded resulting to low BRAF/ pBRAF levels which appeared to interfere with the amplification of apoptotic cell death, previously attained by the combination of TRAIL with 17-AAG (Figure S5).

### BRAFV600E incites rapid DISC formation and engagement of the apoptotic machinery in response to TRAIL

**Figure 6:**
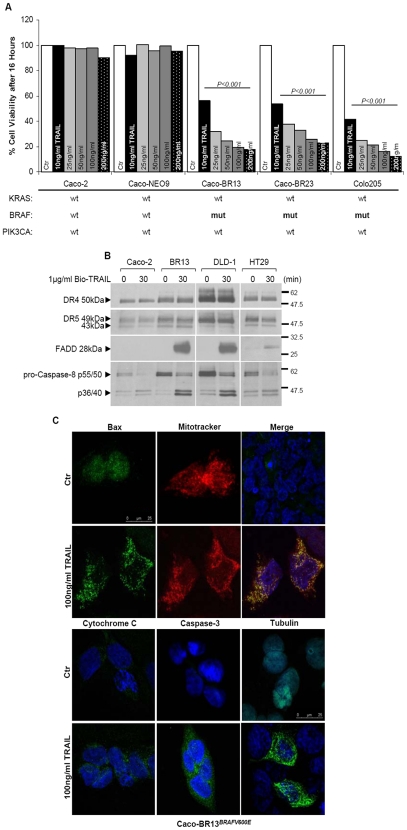
BRAF^V600E^ enhances DISC formation and apoptosis in response to TRAIL in colon cancer cells. (**A**) Cell survival for in Caco-BR and Colo205 cells treated with TRAIL. The values are the average of three independent experiments and are presented as fold change of the absorbance of treated/ untreated cells, for each condition. The cytotoxic effects of TRAIL were assayed by SRB. Treatment conditions are provided along the top on each column. *P<0.05, **P<0.01, ***P<0.001 vs Ctr-untreated. (**B**) DISC complex analysis of functional proteins bound to Bio-TRAIL at indicated time points. (**C**) Rapid induction of apoptosis within 30 minutes with respect to activation of apoptotic markers namely Tubulin, active caspase-3, cytochrome C and Bax were analysed by immunofluorescence after addition of TRAIL. Representative confocal immunofluorescence images, double labelled with Hoechst (blue), Mitotracker (Red) and indicated antibody (green). Original magnification 63x.

To further unravel the role of mutant BRAF as a single mutational event, previously established stable clones overexpressing the mutant protein in a permissive genetic background of Caco-2 cells were analyzed^1^. Significant cell death by apoptosis at the lowest dose of TRAIL was observed in Caco-2 clones overexpressing the mutant BRAF^V600E^ (Caco-BR cells). The extent of apoptosis was comparable to that observed in Colo205 also bearing a single BRAF mutation, but also to that in DLD-1 cells (Figure 6A, Supporting Figure 6).

Analysis of the DISC complex formation in response to TRAIL within 30 minutes indicated that caspase-8 and FADD are recruited to the DISC far more efficiently in the Caco-BR13 and DLD-1 cells than in HT29 cells. In HT29 two mutational pathways are activated, the MAPK and the PI3K pathway, and may be antagonizing each other at the expense of remitting apoptosis (Figure 6B, Supporting Figure 7). Induction kinetics of apoptosis in Caco-BR cells showed that apoptosis was achieved within two hours with the lowest concentration of TRAIL, while only half time treatment with the highest dose was required for efficient induction of apoptosis, as indicated by several key apoptotic markers like proaspase-8 and Bid processing leading to PARP cleavage (Figure S8). Activation of Bax following expeditious mitochondrial release of proapoptotic Cytochrome C, activation of caspase-3 and chromatin condensation within 30 minutes of TRAIL induction was confirmed by immunostaining, correlated to DISC complex formation (Figure 6C).

### PLX4720 requires TRAIL assistance to induce apoptotic cell death in BRAF^V600E^ colon cancer cells

**Figure 7:**
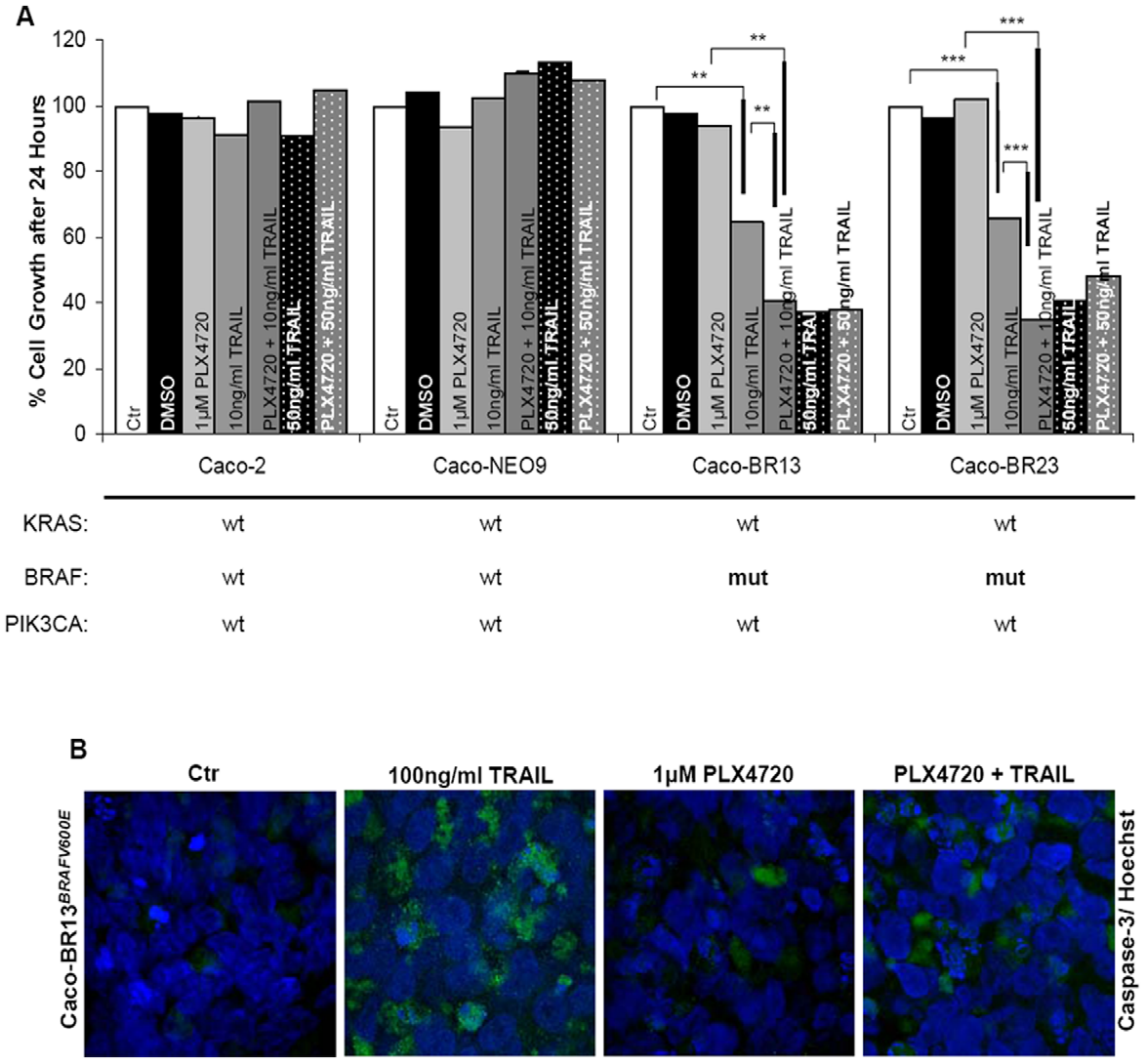
PLX4720 requires TRAIL synergy to induce an anti-proliferative effect. (**A**) Combined treatment with 1 µM PLX4720 for 8 hours and concomitant administration of a low (10 ng/ml) or higher (50 ng/ml) concetration of TRAIL for 16 hours in Caco-BR cells. The values are the average of three independent experiments and are presented as fold change of the absorbance of treated/ untreated cells, for each condition. The cytotoxic effects of TRAIL were assayed by SRB 24 hours after treatment. *P<0.05, **P<0.01, ***P<0.001. (**B**) Caspase-3 activation analysed by immunofluorescence 24 hours after induction with TRAIL/ PLX4720 alone or in combination, in a 3D culture of Caco-BR13 cells. Representative confocal immunofluorescence images, double labelled with Hoechst (blue) and active caspase-3 (green). Original magnification 63x.

As previously observed the presence of mutant BRAF in colon cancer cells, does not predict cell sensitivity to PLX4720 nor did BRAF^V600E^ overexpression in the mutational permissive background of Caco-2 cells (Figure 7A). Limited response to PLX4720 treatment is most likely due to the ambivalence overexpression and phosphorylation of CRAF in response to the mutant BRAF stable induction, but also due to the constitutive activation of MEK/ERK phosphorylation (Figure S9). In order to facilitate cell death, TRAIL was administered in combination with PLX4720. Following exposure to low concentration of TRAIL, cells were sensitized to the combination whereas at higher concentration the advantage was lost due to the conferred sensitivity of BRAF^V600E^ to TRAIL treated cells. Sensitivity of Caco-BR cells to TRAIL and conferred sensitivity following treatment with PLX4720 was also confirmed in 3D culture conditions. Caspase-3 activation and nuclear fragmentation indicated engagement to apoptotic cell death (Figure 7B).

### 17-AAG requires longer incubation periods to induce cell death in the presence of dominant BRAF^V600E^

**Figure 8:**
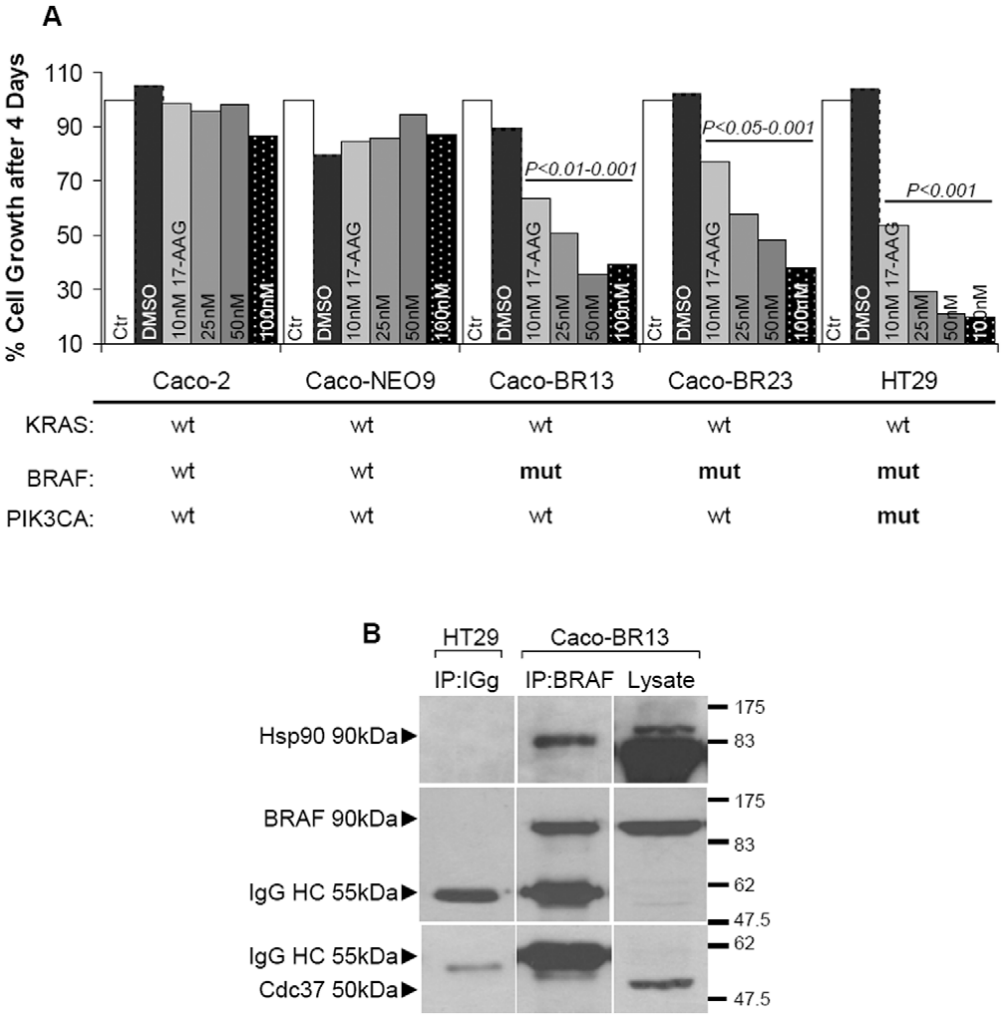
17-AAG requires longer incubation periods to induce cell death. (**A**) Cell survival for Caco-BR and two colon cancer cell lines treated with 17-AAG. The values are the average of three independent experiments and are presented as fold change of the absorbance of treated/ untreated cells, for each condition. The number of viable cells was assayed by SRB 4 days after treatment. Treatment conditions are provided along the top on each column. *P<0.05, **P<0.01, ***P<0.001 vs Ctr-untreated. (**B**) Total cell lysates of Caco-BR13 and HT29 cells were immunoprecipitated with BRAF and the complexes were immunoblotted with Hsp90, BRAF and Cdc37.

The overexpression of mutant BRAF in colon adenocarcinoma cells had quite the opposite effect of the anticipated increase in sensitivity to the 17-AAG inhibitor. Significantly longer (4-day) treatment periods with 17-AAG were required to achieve an antiproliferative effect in Caco-BR cells (Figure 8A) comparable to that achieved in HT29 cells in 24 hours (Figure 2A). Partial degradation of mutant BRAF and insufficient MAPK pathway inhibition in Caco-BR cells overexpressing mutant BRAF^V600E^, appear to preclude cell sensitivity to 17-AAG (Figure S10), regardless of the interaction between mutant BRAF and Hsp90 in the Caco-BR cells (Figure 8B).

### Pathways for *BRAF^V600E^* sensitization of colon cells to TRAIL-induced apoptosis

**Figure 9:**
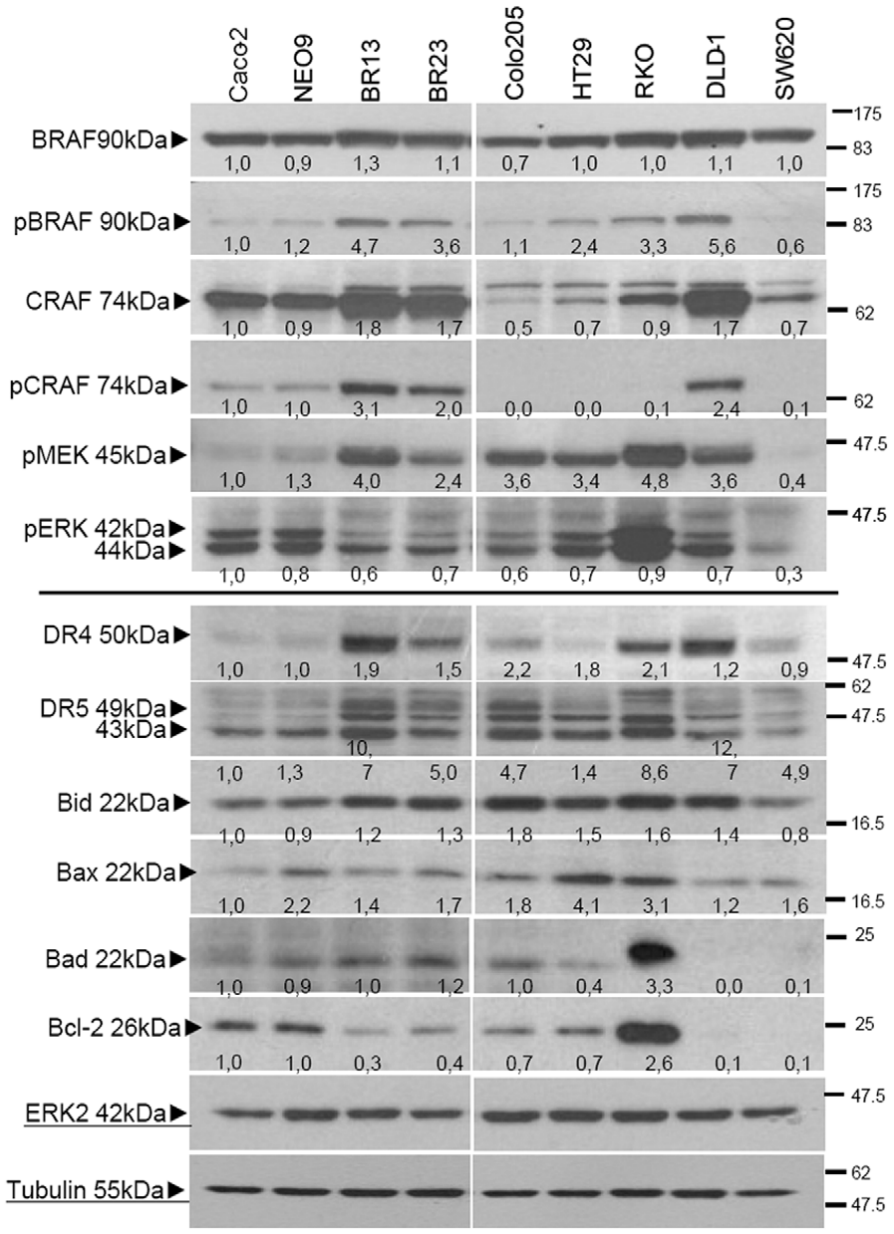
BRAF^V600E^ induces apoptosis related proteins in Caco-2 cells. (**A**) MAPK and apoptotic pathway protein expression analysis in indicated cell lines.

Following mutant BRAF^V600E^ overexpression, the phosphorylation of both BRAF/ CRAF kinases was significantly increased, which resulted in the downstream activation of MEK but not ERK signalling (Figure 9-upper panel, lanes 3–4). Detailed characterisation of this MAPK pathway activation and its cell tumourorigenic effects have been described elsewhere [1]. Increased BRAF/ MEK phosphorylation in Caco-BR cells evoked the differential expression of some key apoptotic and anti-apoptotic molecules, altered in such way that facilitates apoptosis. Increased phosphorylation of the CRAF/ BRAF complex mainly observed in the TRAIL sensitive cell lines Caco-BR and DLD-1 cells correlated with DR4 and DR5 overexpression observed on the cell surface (Figure 9-lower panel, lanes 3-4 and 8; Figure S11). Pro-apoptotic proteins including Bad, Bid and Bax were also found overexpressed in Caco-BR and DLD-1 cells, while anti-apoptotic molecules like Bcl-2 remained unchanged (Figure 9-lower panel, lanes 3–4 and 8). Overexpressed Bad allows Bax to escape Bcl-2 control and drive apoptosis while Bid accumulation caused by BRAF^V600E^ overexpression comprises the initial step towards the sensitization process. Moderate intrinsic pBRAF activity was found in HT29 and RKO cells regardless the presence of a BRAF mutation which was associated with potent activation of the MAPK pathway (Figure 9-upper panel, lanes 6–7). This observation raised the question whether sensitivity to TRAIL induced apoptosis was associated with the constitutive BRAF phosphorylation. Regardless activation of the CRAF/ BRAF complex and TRAIL receptor overexpression in RKO cells (Figure 9-upper panel, lane 7), resistance to TRAIL was recorded (Figure 3A), potentially due to the concomitant activating PIK3CA mutation. Towards this end, we hypothese that the sensitivity of DLD-1 cells to TRAIL is not affected by the concomitant presence of KRAS and PIK3CA mutations because of the constitutive activation of pBRAF in these cells (Figure 9). In contrast, Colo205 cells that did not overexpress the CRAF/ BRAF complex (Figure 9-upper panel, lane 5), but did express only marginally the TRAIL receptors and anti-apoptotic BID (Figure 9-lower panel, lane 5), were successfully committed to apoptosis possibly because of the dominant BRAF^V600E^ presence (Figure 3A).

### Ubiquitination of DR4 following treatment with 17-AAG affects its transcriptional regulation

Significant downregulation of DR4 on the cell surface was observed following treatment with 17-AAG alone or in combination with TRAIL in HT29 cells (Figure 5B-upper panel, lane 2; Figure S12). Immediately a question was raised, regarding the increased apoptosis attained under these conditions. Similar DR4 processing in the presence of 17-AAG was not observed in cells sensitive to 17-AAG inhibitor like DLD-1 and Caco-BR13 cells (Supporting Figures 13, 14). TRAIL alone did not cause any visible proteolysis to either of DRs nor to BRAF/ CRAF expression as compared to the significant degradation of RAF isoforms accompanied by decreased pERK in the presence of 17-AAG (Figure 5B-upper panel, lanes 2–3). The presence of the proteasome inhibitor MG132 managed to suppress 17-AAG-induced BRAF and DR4 degradation in HT29 cells, while CRAF was irreversibly degraded (Figure 5B-upper panel, lane 7). To determine whether DR4 downregulation resulted from decreased pERK, which accompanies BRAF/ CRAF degradation, HT29 cells were treated with the MEK inhibitor UO126 (Figure 5B-upper panel, lane 6) . Inhibition of MEK-ERK signaling resulted in DR4 degradation confirming our model and previous findings, regarding DR been regulated through a MEK-dependent pathway due to increased pERK in the presence of TRAIL and KRAS^G12V^ [17].

**Figure 10:**
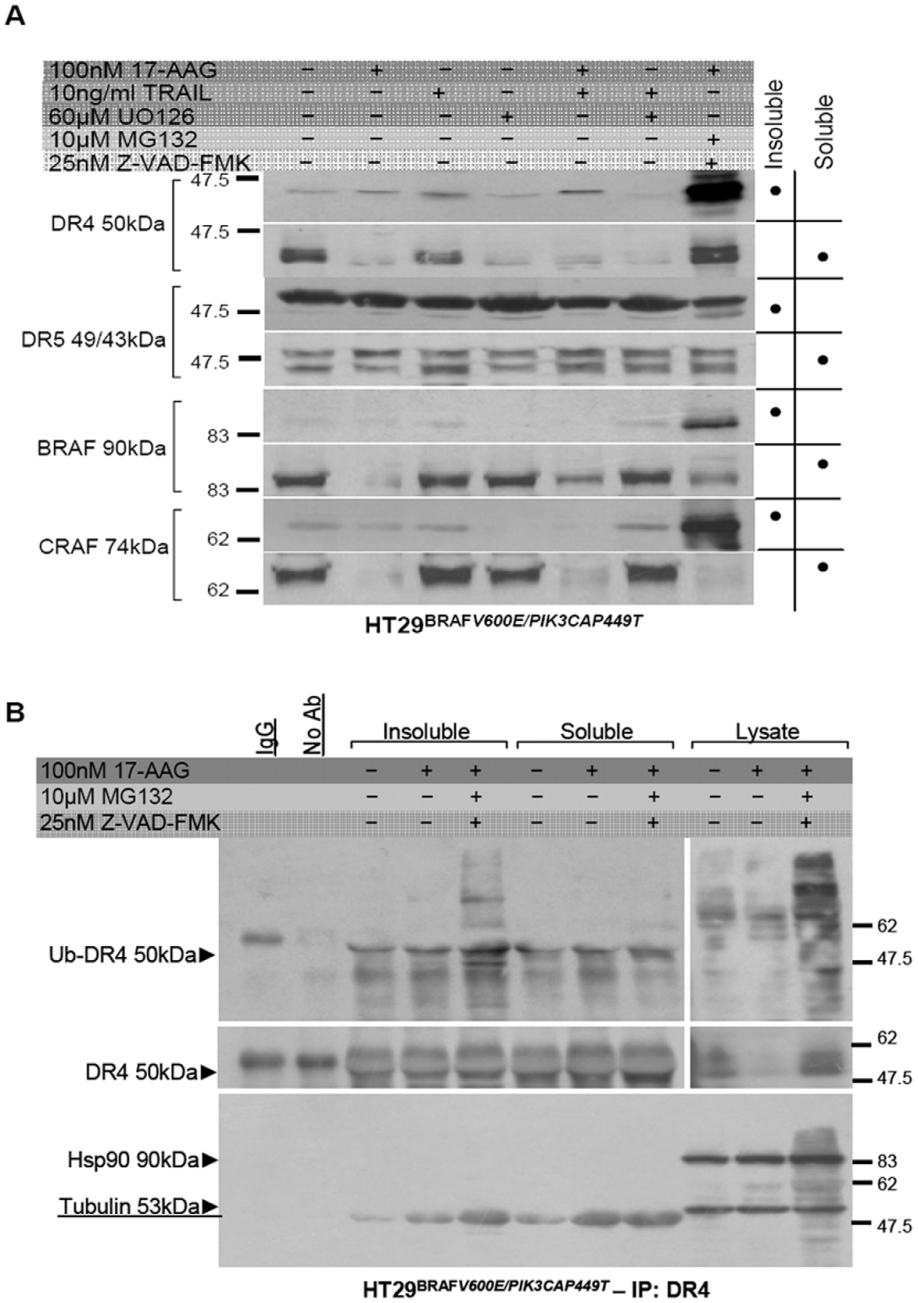
DR4 processing by 17-AAG in HT29 cells. (**A**) NP-40-insoluble fraction from HT29 cell treated as indicated were solubilized in 2% SDS buffer and immunoblotted for the indicated antibodies. The proteasome inhibitor MG132, abrogated 17-AAG-induced loss of BRAF^V600E^ allowing the protected but ubiquitinated BRAF, CRAF and DR4 protein to accumulate in the NP-40 insoluble fraction. (**B**) NP-40 soluble and insoluble fractions were immunoprecipitated with DR4 and blotted with indicated antibodies.

By adopting the double inhibition strategy it was shown that BRAF^V600E^ serves as an Hsp90 client, since a marked increase in RAF isoforms in the NP-40 insoluble fraction was observed (Figure 10A, lane 7). To our surprise DR4 was also detected in the same fraction suggesting that DR4 could also be an Hsp90 client protein targeted for degradation (Figure 10B, lane 7). Even though DR5 was not degraded during 17-AAG treatment, it was also found accumulated in the NP-40 insoluble fraction at all conditions (Figure 10B, lane 7). This is more likely to reflect DRs attached on the cell membrane and dragged into the insoluble. Immunoprecipitation of the insoluble fraction following the double inhibition treatment showed ubiquitin accumulation confirming our hypothesis that DR4 gets ubiquitinated upon treatment with 17-AAG, whereas attempts to co-immunoprecipitate Hsp90 and DR4/5, were unsuccessful (Figure 10B, lane 5). Furthermore, during the double inhibition treatment pERK activity remained suppressed in the cytosolic extracts, while DR4 was rescued. This should not negate MEK-ERK dependent regulation of DR4, since proteins found in an ubiquitinated state should have their signaling ability impaired, which is depicted by the selective mRNA downregulation of DR4 at these conditions (Figure S15). Nevertheless, regardless complete absence of DR4 in HT29 cells, apoptosis was amplified in the combined treatment via the DR5.

### 17-AAG sensitizes HT29 cells to TRAIL-induced apoptosis via DR5

**Figure 11:**
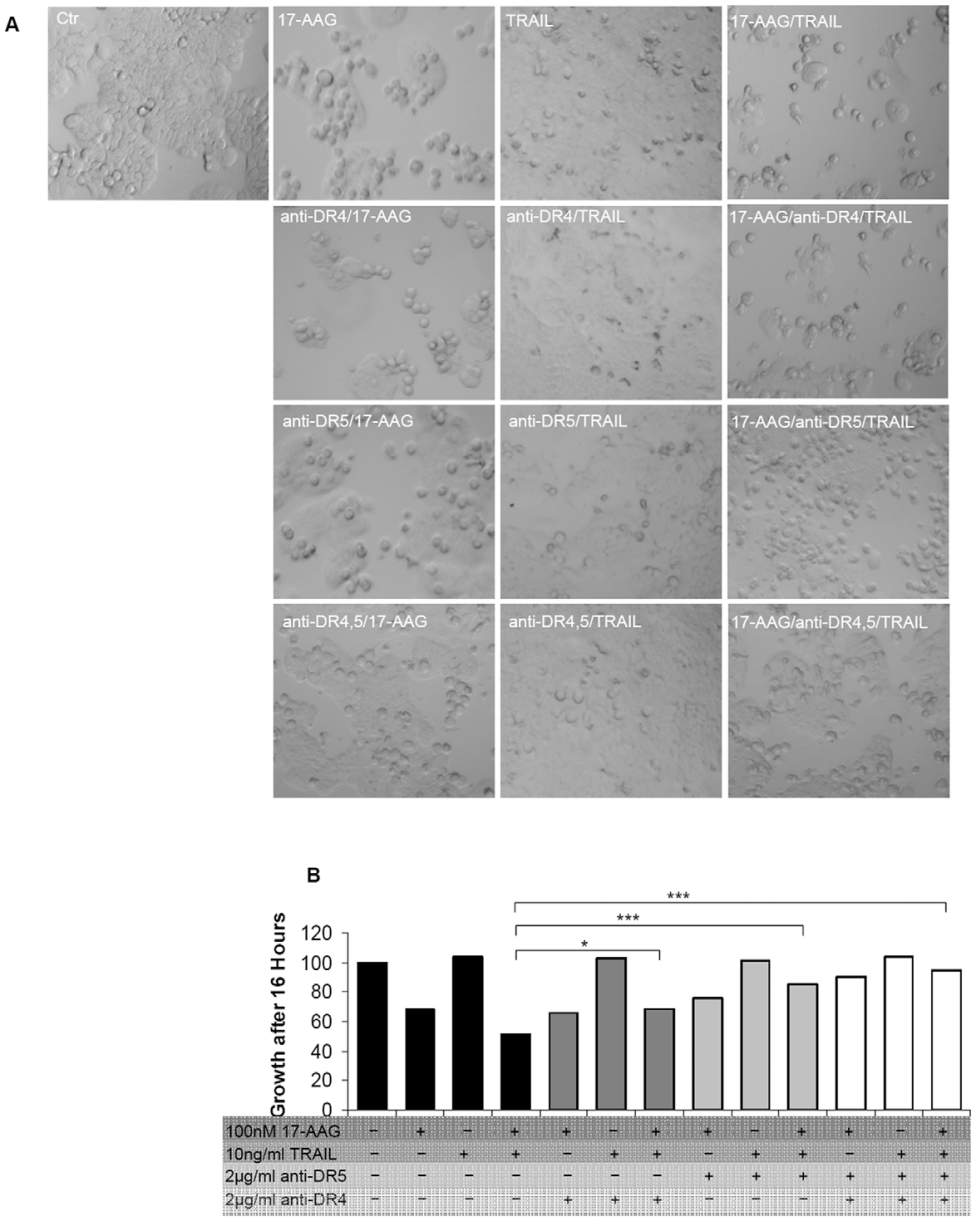
17-AAG sensitizes HT29 cells to TRAIL-induced apoptosis via the DR5. (**A**) Effects of anti-DR4/DR5 blocking antibodies on TRAIL/17-AAG combination treatment in HT29 cells. Pre-treatment for 15 minutes with blocking antibody preceded all indicated treatments. Representative images, original magnification 30x. (**B**) Cell viability of HT29 cells assays by SRB. Cell viability is presented as fold change of the absorbance of treated/ untreated cells, for each condition. *P<0.05, **P<0.01, ***P<0.001.

To prove our hypothesis regarding DR5 being the mediator of apoptosis following treatment with 17-AAG, HT29 cells were pre-treated with a blocking antibody against DR4 or DR5 and then subjected to TRAIL treatment. Inhibition of cell death was achieved in the presence of the blocking Ab against DR5 but not against DR4, regardless TRAIL been administered alone or in combination with 17-AAG. In addition, inhibition of cell death was also achieved when both blocking antibodies were combined during pretreatment (Figure 11A-B). Data obtained so far suggest that increased apoptosis in HT29 cells after combined treatment of 17-AAG with TRAIL is mediated via DR5.

## Discussion

Constitutive activation of signaling via EGFR, MAPK and PI3K pathways can promote uncontrolled cell growth, tumor cell survival as well as resistance to cytotoxic agents [28], [29]. Inhibiting the source of activation, when due to activating mutations (KRAS, BRAF, PI3K), is a complex task. Inhibition of mutant BRAF^V600E^ using the selective PLX4720 inhibitor, induces apoptosis in melanoma cells [18]. In contrast, the clinical activity seen with PLX4032 in metastatic CRC was more modest suggesting the biology behind this type of cancer is more heterogeneous [30]. Initiation and progression of CRC proceeds through a series of genetic alterations involving oncogenes and tumour suppressor genes, therefore prolonged and substantial suppression can only be achieved in combination therapy. In the present study we present the anti-proliferative effects of the pharmacological application of PLX4720 as a single agent or in combination with TRAIL, an inducer of apoptosis. Treatment resistance recorded after exposure to PLX4720, had not been anticipated considering studies reporting that BRAF mutant cancers are very sensitive to pharmacological MEK inhibitors and did not correlate to the mutational background of the cells [31]. In our study, even though inhibition of cell proliferation was not accomplished by PLX4720, successful inhibition of downstream targets pMEK/ pERK with a profound effect on cyclin D1 target gene was achieved in much shorter incubation periods indicating a small therapeutic window. This observation illustrates the need for supportive combinatorial treatment. The proliferative advantage previously described in cells bearing KRAS mutant and wild-type BRAF following treatment with PLX4720, was only observed for SW620 cells. In contrast, cell numbers of DLD-1 cells remained unchanged suggesting a possible implication of constitutive pBRAF in these cells. Towards this end, when PLX4720 was co-administered with TRAIL, in TRAIL sensitive DLD-1 cells, TRAIL induced apoptosis was abrogated indicating wild-type BRAF and not mutant KRAS, also present in DLD-1, as the exclusionary factor for colon cancer treatment with PLX4720.

Pharmacological application of an indirect BRAF^V600E^ inhibitor, 17-AAG as a single agent was more efficient in colon cancer cells. The antiproliferative effect was not selective to a specific mutational background but to the levels of AKT phosphorylation. Treatment with 17-AAG was least effective in RKO cell line, where an increased phosphorylation of AKT is observed. Notably, recent studies have shown that PIK3CA^MT^ and altered pAKT activity can be critical markers for optimal tumour treatment protocols [32], [33]. Most encouraging was the selectively of 17-AAG towards SW620, a metastatic colon cancer cell line, both Fas and TRAIL-resistant [34]. Antiproliferative treatment with PLX4720 could not be implemented within the small therapeutic window predicted and the 17-AAG inhibitor could not eradicate tumour cells under short time exposure at low concentration, their combination with an apoptotic agent like TRAIL proved essential and could intensify the results on cancer cell death. Thus, whether the clinical goal is long-term exposure to low concentrations or intermittent administration of relatively higher doses, the present study proposes rational drug combinations and their therapeutic application that appears to offer superior opportunities provided its tolerability in humans is acceptable, which will require phase I clinical evaluation.

In order to achieve rapid induction of apoptosis, combination of tested BRAF inhibitors with TRAIL in a concomitant approach was applied in cell lines exhibiting some degree of sensitivity to these inhibitors. When TRAIL was applied as a single agent conferred limited cytotoxity in colon cells harboring activating mutations in both the MAPK and PI3K pathways, whereas its effect was significantly increased in cells with a single BRAF mutation and accompanied by high pBRAF levels. If the presence of dominant BRAF^V600E^ and high presentation of pBRAF serve as a sensitizing factor to TRAIL induced apoptosis, then the significantly activate wild-type BRAF in DLD-1 cell may also be a TRAIL sensitizer. The combination of PLX4720 with concomitant TRAIL induction managed to increase cell death by apoptosis in RKO cells but not in HT29, even though downstream targets like pMEK/ pERK levels were equally inhibited. Interestingly, the presence of PIK3CA mutation and the highly activated PI3K/Akt pathway in RKO cells does not inhibit the synergism between PLX4720 and TRAIL, which can be further exploited in the clinical application of these pharmacologic agents. The importance of this finding is further emphasized by the fact that pharmacologic MEK pathway inhibition of cell proliferation in BRAF mutant cancers may be significantly decreased by the presence of activating PIK3CA mutations [35].

In order to identify molecular modifiers that may influence cell sensitivity to apoptosis even further, the proposed role of mutant PIK3CA as an inhibitory factor was explored in our study [35]. Inhibitors of the PI3K/Akt signaling pathway gain considerable attention in the treatment of CRC, especially since PI3K/Akt pathway inhibition can modulate TRAIL induced apoptosis in HT29 cells [34]. Down-regulation of PI3K by siRNA may also sensitize colon cancer cells to TRAIL-induced apoptosis [36]. Herein, in order to improve cell response to BRAF inhibitor treatment, attenuation of the PIK3CA^H1047^ that co-exist with BRAF^V600E^ in the relevant colon cancer cell lines was performed. Deletion of PI3K using the PI-103 inhibitor alone or in combination with either PLX4720 or TRAIL ranged from inactive to moderate respectively and was only marginally increased when PI-103 was incorporated into the PLX4720/ TRAIL combination in RKO cells. In conclusion, the presence of PI3KCA mutation might not be interfering with the anti-proliferative action of the PLX4720/ TRAIL combination, but its suppression might offer an additional advantage towards apoptotic cell death. Replacing PI-103 inhibitor with an siRNA against PIK3CA^H1047^ in RKO cells contributed equally to the induction of apoptosis, illustrating once again the inhibitory role of PIK3CA^H1047^ as well as the benefits of using siRNA as a treatment alternative. The combination efficacy of BRAF inhibition and TRAIL treatment in the 2D cell culture system was also validated in three-dimensional spheroid culture conditions, a pre-clinical experimental model that may indicate the most promising combinations to be later exploited in the clinic.

In a similar approach the PI-103 inhibitor was also used in combination with TRAIL in HT29 cells. Increased sensitivity to apoptosis was evident but there were no signs of further synergism when incorporated into the PLX4720/ TRAIL combination. The PI3K inhibitor PI-103 has been shown to efficiently cooperate with TRAIL to synergistically induce apoptosis [37]. When PLX4720 was replaced by shRNA against BRAF^V600E^ in HT29 cells, there was not additive effect on apoptotic cell death for any of the treatment combinations, suggesting that the shRNA approach in a heterozygous mutant background is not very efficient. If our earlier hypothesis identifying activated-mutated BRAF as a sensitizing factor is valid, the significantly low pBRAF activity present in HT29 cells, as compared to RKO and DLD-1, may explain the semi-resistant response not only to TRAIL but also to the different combinations applied. In addition to the moderate pBRAF activation, we also noted that the PIK3CA^P449T^ activating mutation in HT29 is also of low activity which suggests that none of the MAPK or the PI3K pathways is dominant. A simulated balance created by both pathways prevents the cell from shifting completely into the apoptotic pathway, achieving as a result mild sensitization to apoptosis.

The synergistic effect between TRAIL and 17-AAG led to a significantly apoptotic response in HT29 cells. Induction of apoptosis was most likely achieved through amplification of the mitochondrial pathway based on the extensive pro-caspase-9 processing in the combined treatment. It has been previously demonstrated that combination of TRAIL with certain signaling inhibitors results in enhancement of apoptosis through inhibition of important pro-survival pathway components like AKT [38], [39]. Activated AKT kinase is known to be an Hsp90 client and can be targeted by 17-AAG [23]. Nevertheless, the anti-proliferative effect of 17-AAG alone or in combination with TRAIL observed in HT29 and DLD- 1 cells relays entirely on the interaction between Hsp90 and BRAF either mutated or activated by KRAS^G13D^ respectively, since depletion of mutant BRAF^V600E^ in HT29 resulted to complete reversal of apoptotic cell death. Interaction between Hsp90 and mutant BRAF is impaired resulting to even lower pBRAF activity that interferes with the amplification of apoptotic cell death.

To further unravel the role of mutant BRAF as a single mutational event, a stable expression of BRAF^V600E^ was performed in Caco-2 cells. Overexpression of the mutant protein significantly sensitized Caco-2 cells to TRAIL induced apoptosis, a response comparable to that of Colo205 cells also bearing a single BRAF mutation. Functional analysis of DRs and apoptosis kinetics revealed rapid DISC assembly and activation of the mitochondria-dependent pathway after induction with TRAIL in BRAF^V600E^ transformed and high pBRAF presenting DLD-1 cells. Nevertheless, overexpression of mutant BRAF does not confer any sensitivity to the antiproliferative effect of PLX4720, unless a small concentration of TRAIL is added to achieve an additive effective response. Limited response to PLX4720 treatment is most likely due to the ambivalence overexpression and phosphorylation of CRAF in response to mutant BRAF stable induction. The successful combination of PLX4720 with TRAIL in cells overexpressing mutant BRAF was also confirmed in three-dimensional culture conditions. Efficacy of TRAIL in Caco-BR cells grown in spheroids holds great potential for future application, especially when apoptosis is engaged in microsatellite instable Caco-BR cells [1], potentially by circumventing their impaired DNA repair mechanism in addition to deregulated signaling pathways. The deregulate cell cycle Caco-BR cells are under not only because of their deregulation of their mitotic check point [1], but also because of the high levels of hyperphosphorylated cyclinD1, might be accounted for the very late response of the Caco-BR clones to 17-AAG treatment. The unstable cell cycle renders Caco-BR cells able to escape 17-AAG cell cycle targeted effects. On the other hand, HT29 cells have a more stable cell cycle that can be more efficiently targeted by 17-AAG, whereas DLD-1 cells harboring a KRAS mutation were also found partially sensitive to 17-AAG, possibly because of their high intrinsic pBRAF levels. Sensitivity of cancer cells to specific drugs can be regulated through the expression patterns of the BH3-only family members.

Overexpression of pro-apoptotic as compared to anti-apoptotic factors was recorded following BRAF^V600E^ transformation and was comparable to high pBRAF presenting DLD-1 and Colo205 cells. Recorded upregulation in DR4/ 5 serve as the initial step towards the efficient DISC formation whereas Bid accumulation provides the link between terminal effector processes and signaling related alterations such as DNA integrity, cell attachment and microtubule function. Simultaneous downregulation of Bcl-2 ensures Bax translocation to the mitochondrial membrane and Cytochrome C release. Upregulation of pro-apoptotic proteins may explain how increased sensitivity of Caco-BR cells to TRAIL-induced apoptosis is achieved.

Previous studies describe the synergistic effect between TRAIL and 17-AAG and the inhibitory effect of 17-AAG on DR4. Although it has been well described how the ubiquitin system regulates the proximal steps of the DISC assembly and its components, ubiquitination of DR4 has not been observed before [40]. Despite ubiquitin dependent proteolysis of DR4 accompanied by an mRNA depended downregulation, lack of interaction between Hsp90 and DR4 suggests that DR4 is targeted by the ubiquitin aimed for HSp90. In this case internalization of DR4 must be more frequent as compared to DR5 or the protein turnover is increased. Moreover, significant suppression of pERK following BRAF degradation by 17-AAG has also contributed to a MEK dependent DR4 downregulation in the presence of attenuated BRAF^V600E^ activity. Nevertheless, apoptosis was efficiently mediated through DR5 as determined by functional analysis using specific DR5 blocking antibodies. Considering occasions where inactivation of KILLER/ DR5 due to mutations [41], or absence of its cell surface expression because of improper transport to the cell surface [42], cells being treated with the proposed combination of TRAIL/ 17-AAG will manage to escape DR5 mediated apoptosis.

This study has analyzed in detail the role of BRAF/ KRAS/ PIK3CA mutation status of particular colorectal tumours on predicting efficient therapeutic treatments with the BRAF and the PI3K inhibitors as well as their rational combination with TRAIL. Cell death by apoptosis when TRAIL was concomitantly administered with PLX4720 or 17-AAG was significantly increased suggesting that when BRAF^V600E^ or PIK3CA^H1047^ exist as dominant mutational events may confer sensitivity to TRAIL.

## Supporting Information

Figure S1 **Suppression of BRAF phosphorylation within 16 hours and rapid inhibition of pERK within an hour of treatment with 1 and 10 µM of PLX4720 in RKO cells.**
(TIF)

Figure S2 **Complete experiment of immunoprecipitation of indicated cell lines with BRAF and the complexes subsequently immunoblotted first with Hsp90 and then with BRAF and Cdc37, which is right bellow the heavy chain (HC) of the antibody.**
(TIF)

Figure S3 **Combined treatment with 1 µM PLX4720 for 8 hours and concomitant administration of 10 ng/ml TRAIL for 16 hours in DLD-1 and HCT116 colon cancer cells.** Cell viability assayed by SRB 24 hours after treatment. **P<0.01, ***P<0.001.
(TIF)

Figure S4 **Depletion of mutant BRAF by transient transfection using indicated shRNA against the BRAF^V600E^ present in HT29 cells and subsequent treatment of cells with 100 ng/ml TRAIL.** Total cell lysates harvested for the described treatments were immunoblotted with the indicate antibodies.
(TIF)

Figure S5 **(B) Western blotting of HT29, HT-PS (empty vector), HTShBR-1, -3 and -5 stable clones.** Expression levels of total and phosphorylated BRAF is shown accompanied by phosphorylation status of ERK1/2.
(TIF)

Figure S6 **A dose response with TRAIL was performed for 16 hours in Caco-BR transformed cells and BRAF^V600E^ mutant HT29 cells.** The cytotoxic effects of TRAIL were measured using the apoptosis ELISA kit by Roche. Log percentage cell viability and fold change of the absorbance of treated/ untreated cells, for each condition are presented.
(TIF)

Figure S7 **Cell lysates and flow through controls of the western blot analysis for the DISC immunoprecipitation.**
(TIF)

Figure S8 **Induction kinetics of apoptosis at indicated TRAIL concentrations and specific time points.** Total cell lysates harvested from cells treated for indicated time points following treatment with 10 and 100 ng/ml TRAIL were immunoblotted for the indicated antibodies. Proteins are quantified against α-tubulin.
(TIF)

Figure S9 **Total cell lysates harvested from in Caco-BR cells treated for 4 hours with indicated concentrations of PLX4720 were immunoblotted for the indicated target proteins.** Proteins are quantified against α-tubulin.
(TIF)

Figure S10 **Total cell lysates harvested from Caco-BR13 cells treated for indicated time points with 100 nM 17-AAG were immunoblotted for the indicated antibodies.** Proteins are quantified against α-tubulin.
(TIF)

Figure S11 **Cell surface expression of DR4 and DR5 analysed by means of flow cytometry following staining with antibodies against DR4, DR5 and the secondary GAM-PE antibody only (IgG-PE) that was used against DR4 and DR5 in living cells (Hoechst negative).** Blue line indicates expression levels of DRs in parental Caco-2 cells. Representative histograms from at least three independent experiments are shown. Blue line indicates expression levels of DRs in parental Caco-2 cells.
(TIF)

Figure S12 **DR expression on the cell surface of HT29 cells.** Downregulation of the DR4 was assayed by means of flow cytometry following treatment with 100 nM 17-AAG alone or in combination with 10 ng/ml TRAIL for 24 hours. Nearly 40% of the DR4 was rescued in the presence of 10 µM MG132. Representative histograms from at least four independent experiments are shown. Blue line indicates expression levels of DRs in parental Caco-2 cells.
(TIF)

Figure S13 **DR4 escapes 17-AAG dependent degradation in DLD-1 cells.** Cells were left untreated or treated with 150 nM or 1 µM 17-AAG for 30 hours, or pre-treated with the 150 nM 17-AAG for 24 hours after which 10 ng/ml TRAIL was added for another 16 hours or 10 ng/ml TRAIL alone was added for 16 hours. Alternatively cells were pre-treated with 25 nM Z-VAD-FMK for 1 hour then 10 µM MG132 was added for another hour after which cell were treated with 150 nM 17-AAG for 24 hours. Protein extracts were separated into NP-40 soluble and NP-40 insoluble fractions and subjected to Western blot analysis.
(TIF)

Figure S14 **Caco-BR13 cells were left untreated or treated with a low 150 nM and a high 1 µM concentration of 17-AAG for 30 hours, or pre-treated with the 1 µM 17-AAG for 24 hours.** Alternatively cells were pre-treated with 25 nM Z-VAD-FMK for 1 hour then 10 µM MG132 was added for another hour after which cell were treated with 1 µM 17-AAG for 24 hours. Protein extracts were subjected to Western blot analysis. Picture shown is representative of three independent experiments.
(TIF)

Figure S15 **mRNA extracts from HT29 cells treated as indicated were analysis by RT-PCR with regard to TRAIL receptor, DR4 and DR5, transcriptional activity.**
(TIF)

File S1 **Supplementary** **Materials and Methods** **.**
(DOC)
