# Supplementary figures and images for: Selective BRAFV600E Inhibitor PLX4720, Requires TRAIL Assistance to Overcome Oncogenic PIK3CA Resistance
Source: PLoS One. 2011 Jun 27;6(6):e21632. doi: 10.1371/journal.pone.0021632 (PMC3124547; doi:10.1371/journal.pone.0021632)

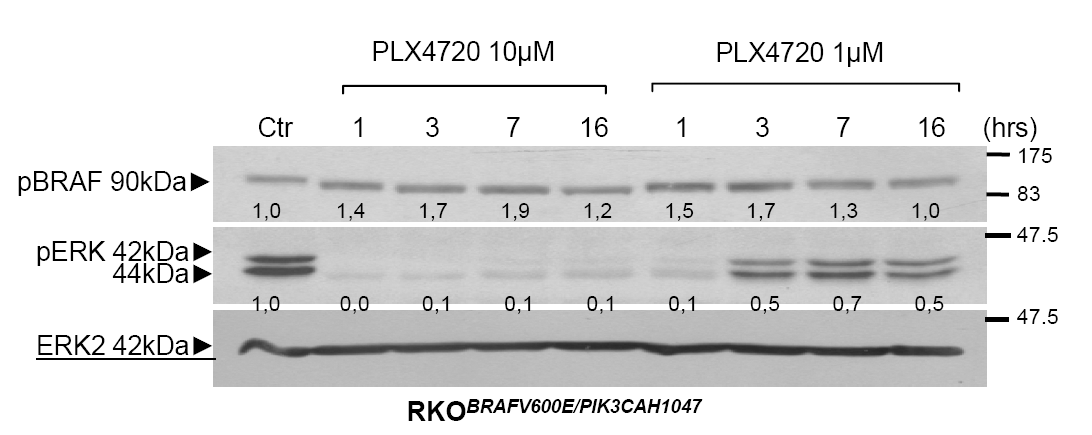

Supplement: Figure S1 — Suppression of BRAF phosphorylation within 16 hours and rapid inhibition of pERK within an hour of treatment with 1 and 10 µM of PLX4720 in RKO cells. (TIF) [file pone.0021632.s001.tif]

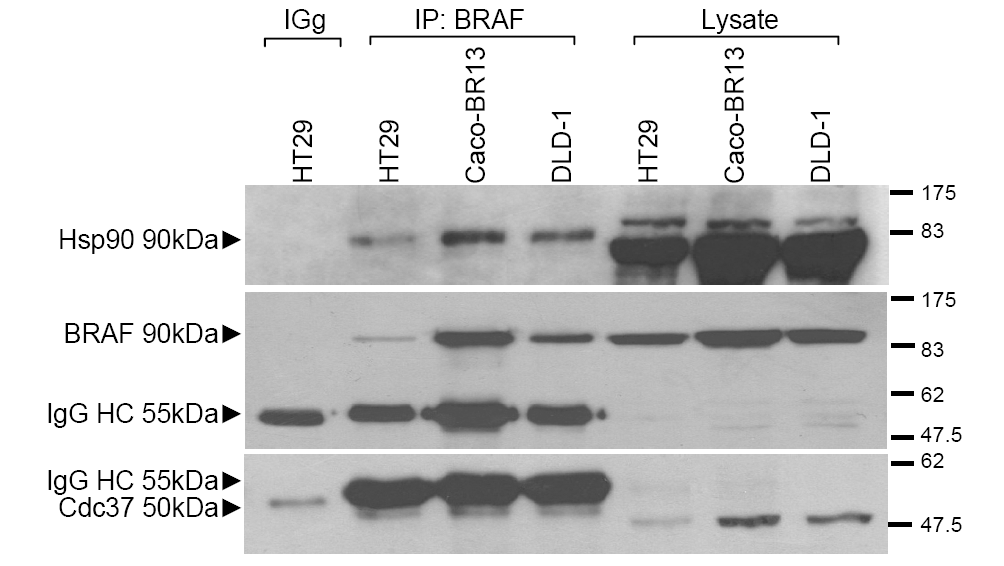

Supplement: Figure S2 — Complete experiment of immunoprecipitation of indicated cell lines with BRAF and the complexes subsequently immunoblotted first with Hsp90 and then with BRAF and Cdc37, which is right bellow the heavy chain (HC) of the antibody. (TIF) [file pone.0021632.s002.tif]

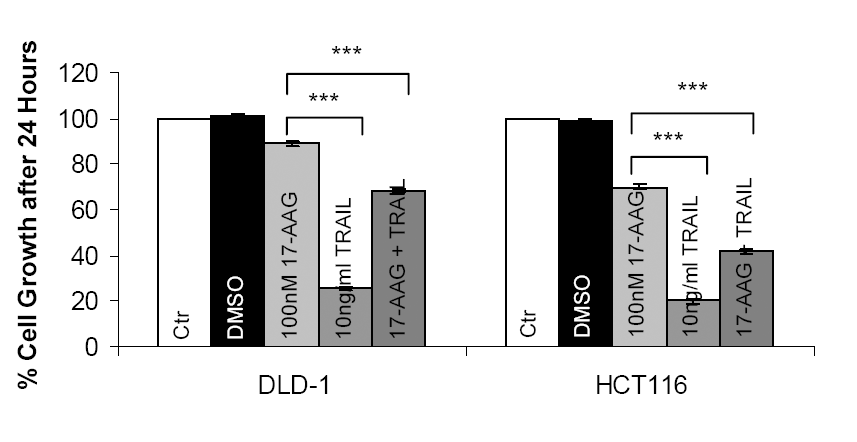

Supplement: Figure S3 — Combined treatment with 1 µM PLX4720 for 8 hours and concomitant administration of 10 ng/ml TRAIL for 16 hours in DLD-1 and HCT116 colon cancer cells. Cell viability assayed by SRB 24 hours after treatment. **P<0.01, ***P<0.001. (TIF) [file pone.0021632.s003.tif]

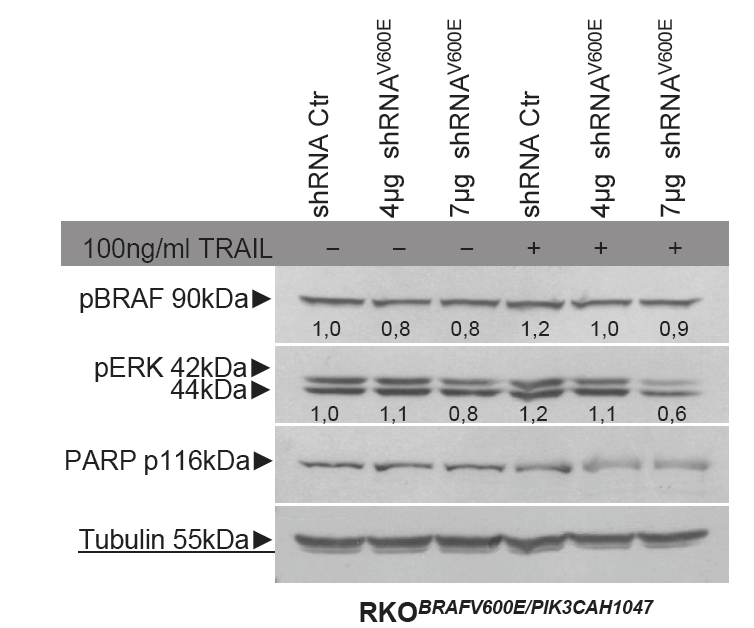

Supplement: Figure S4 — Depletion of mutant BRAF by transient transfection using indicated shRNA against the BRAFV600E present in HT29 cells and subsequent treatment of cells with 100 ng/ml TRAIL. Total cell lysates harvested for the described treatments were immunoblotted with the indicate antibodies. (TIF) [file pone.0021632.s004.tif]

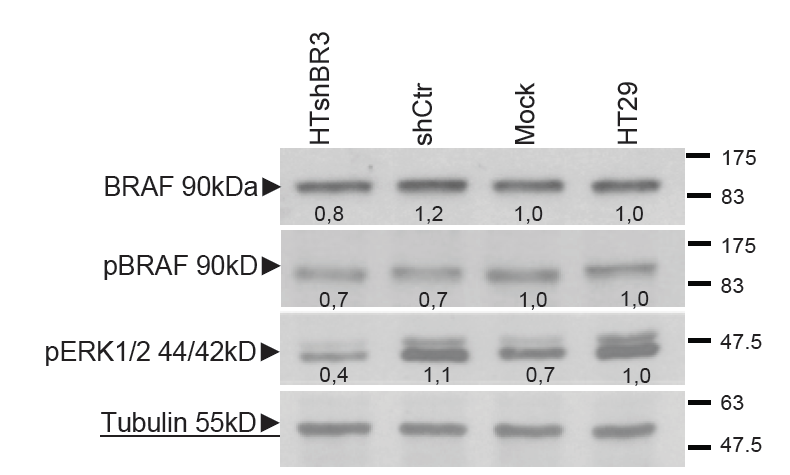

Supplement: Figure S5 — (B) Western blotting of HT29, HT-PS (empty vector), HTShBR-1, -3 and -5 stable clones. Expression levels of total and phosphorylated BRAF is shown accompanied by phosphorylation status of ERK1/2. (TIF) [file pone.0021632.s005.tif]

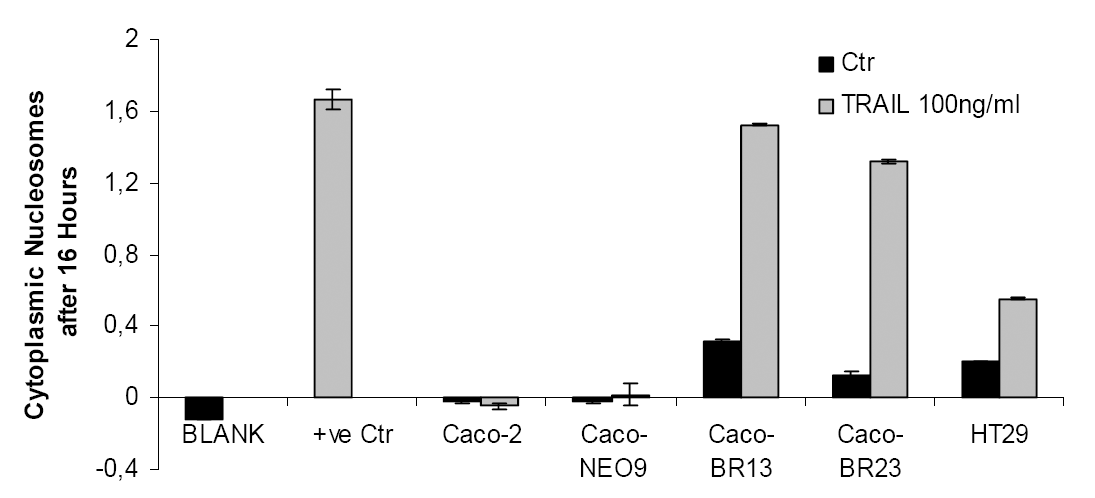

Supplement: Figure S6 — A dose response with TRAIL was performed for 16 hours in Caco-BR transformed cells and BRAFV600E mutant HT29 cells. The cytotoxic effects of TRAIL were measured using the apoptosis ELISA kit by Roche. Log percentage cell viability and fold change of the absorbance of treated/ untreated cells, for each condition are presented. (TIF) [file pone.0021632.s006.tif]

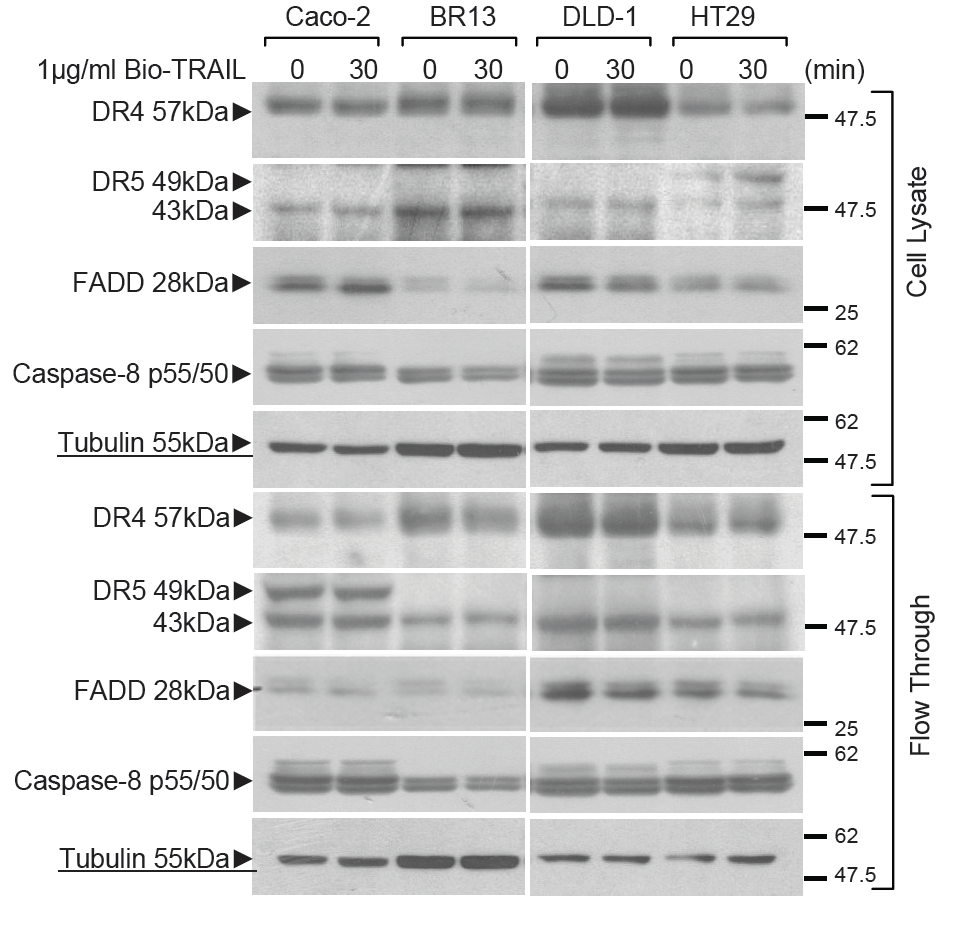

Supplement: Figure S7 — Cell lysates and flow through controls of the western blot analysis for the DISC immunoprecipitation. (TIF) [file pone.0021632.s007.tif]

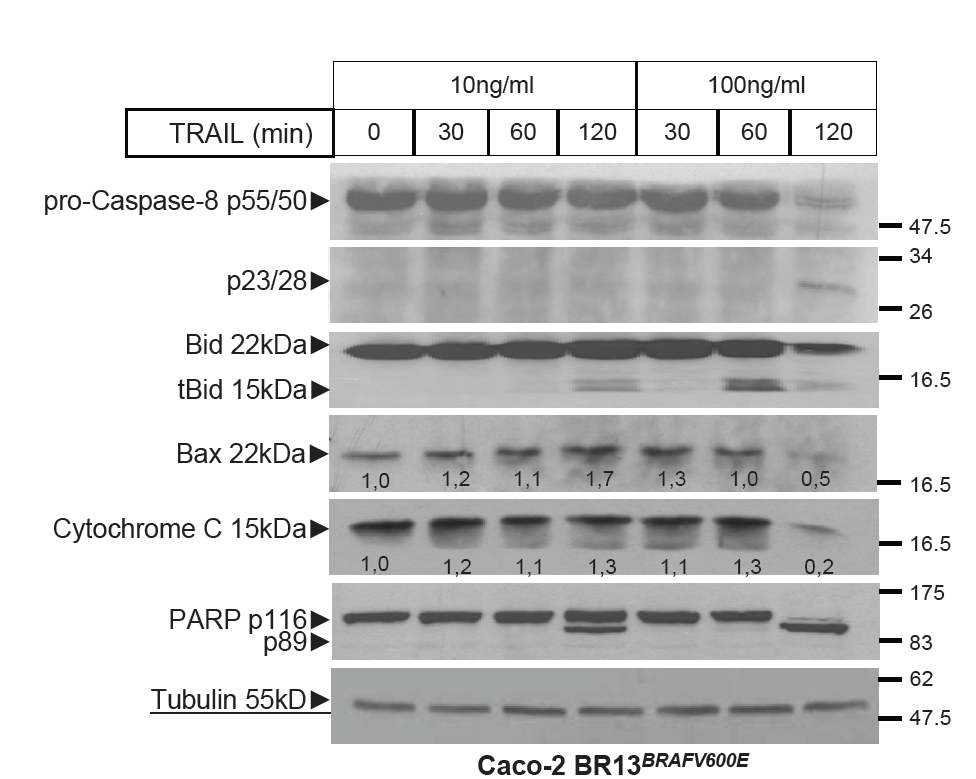

Supplement: Figure S8 — Induction kinetics of apoptosis at indicated TRAIL concentrations and specific time points. Total cell lysates harvested from cells treated for indicated time points following treatment with 10 and 100 ng/ml TRAIL were immunoblotted for the indicated antibodies. Proteins are quantified against α-tubulin. (TIF) [file pone.0021632.s008.tif]

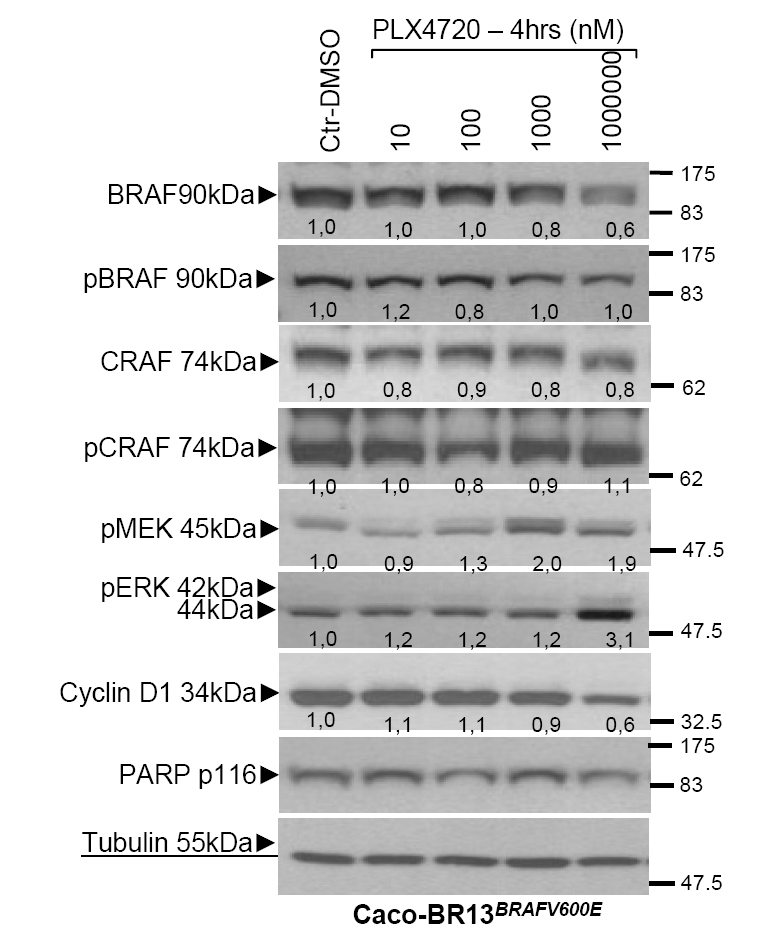

Supplement: Figure S9 — Total cell lysates harvested from in Caco-BR cells treated for 4 hours with indicated concentrations of PLX4720 were immunoblotted for the indicated target proteins. Proteins are quantified against α-tubulin. (TIF) [file pone.0021632.s009.tif]

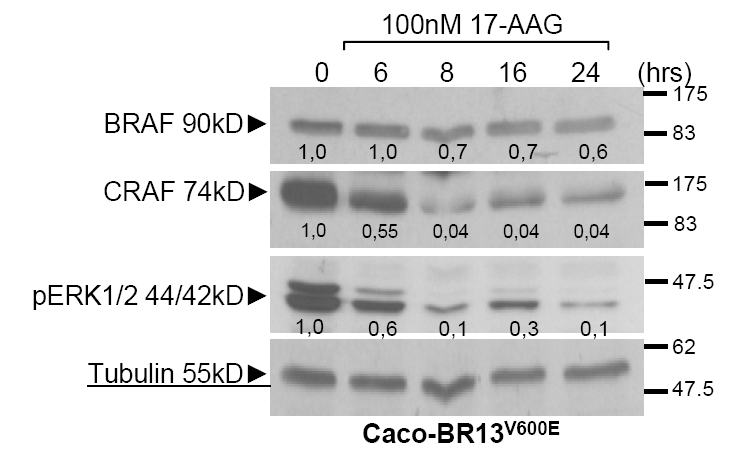

Supplement: Figure S10 — Total cell lysates harvested from Caco-BR13 cells treated for indicated time points with 100 nM 17-AAG were immunoblotted for the indicated antibodies. Proteins are quantified against α-tubulin. (TIF) [file pone.0021632.s010.tif]

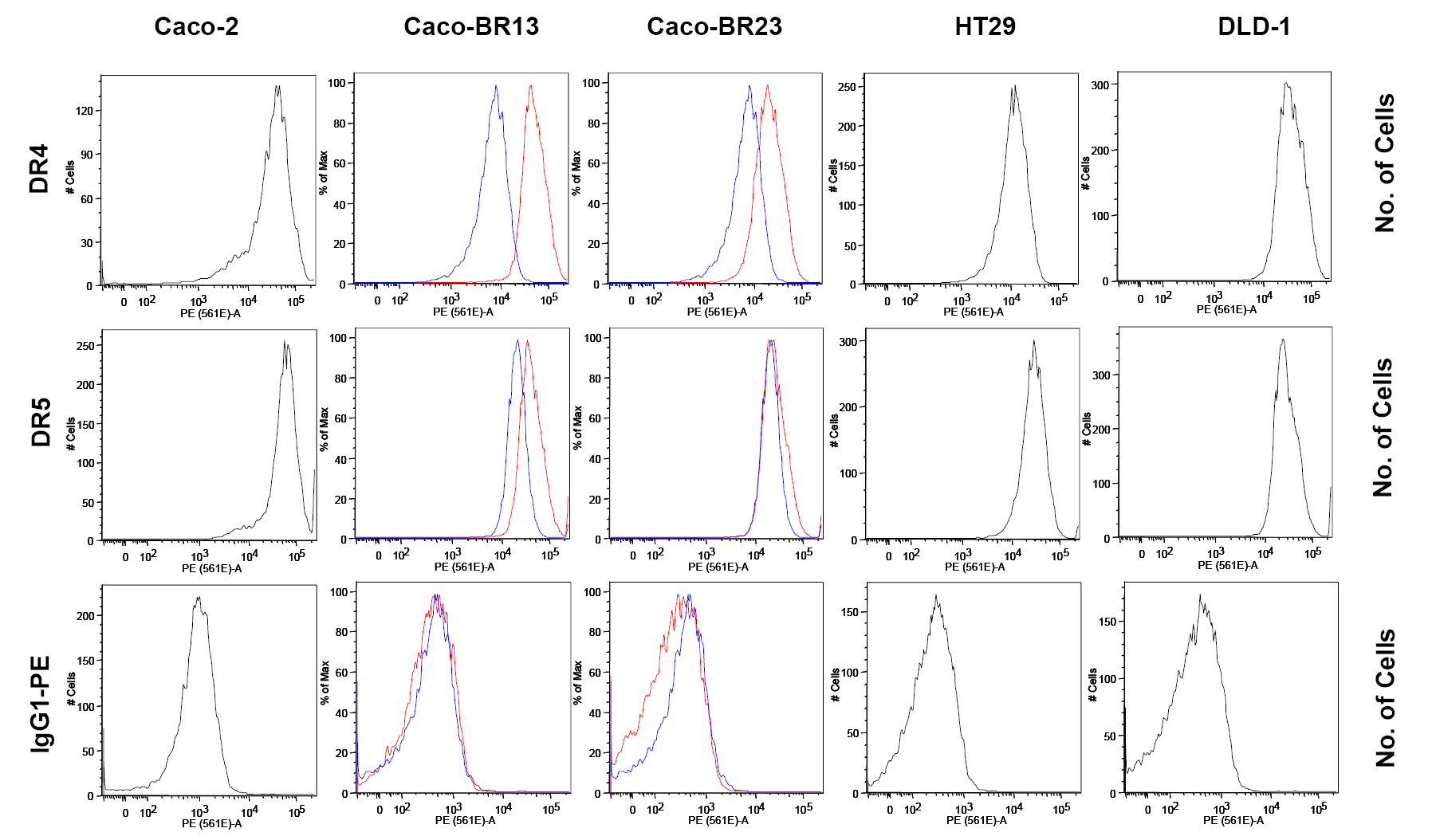

Supplement: Figure S11 — Cell surface expression of DR4 and DR5 analysed by means of flow cytometry following staining with antibodies against DR4, DR5 and the secondary GAM-PE antibody only (IgG-PE) that was used against DR4 and DR5 in living cells (Hoechst negative). Blue line indicates expression levels of DRs in parental Caco-2 cells. Representative histograms from at least three independent experiments are shown. Blue line indicates expression levels of DRs in parental Caco-2 cells. (TIF) [file pone.0021632.s011.tif]

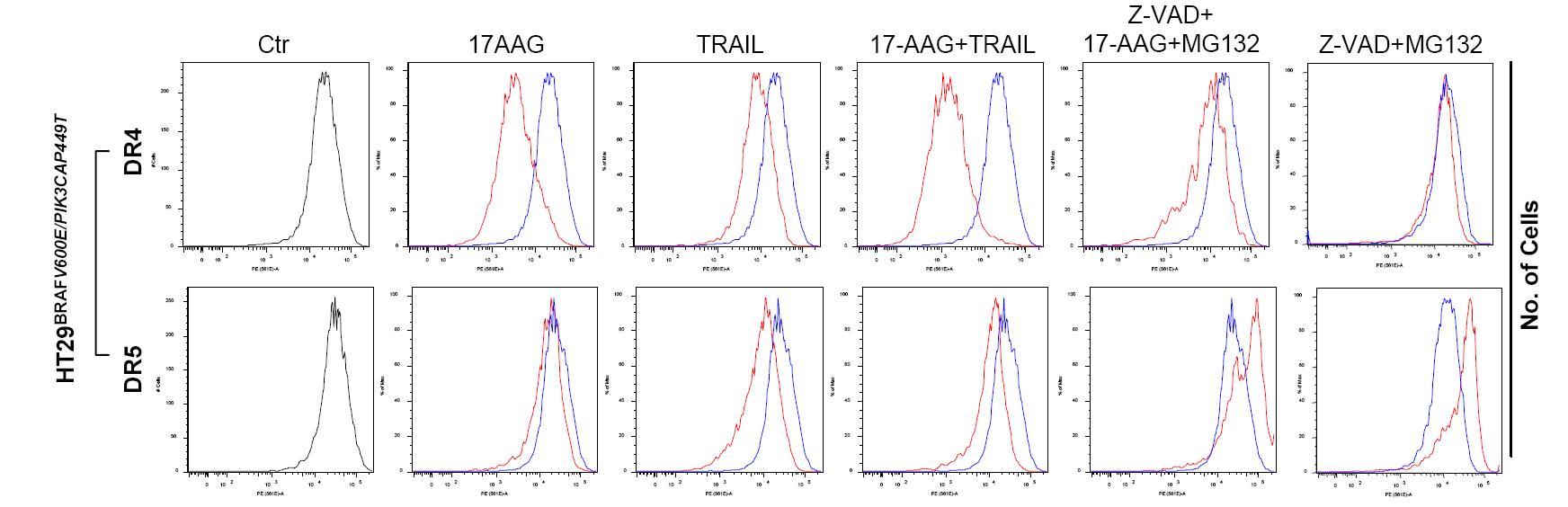

Supplement: Figure S12 — DR expression on the cell surface of HT29 cells. Downregulation of the DR4 was assayed by means of flow cytometry following treatment with 100 nM 17-AAG alone or in combination with 10 ng/ml TRAIL for 24 hours. Nearly 40% of the DR4 was rescued in the presence of 10 µM MG132. Representative histograms from at least four independent experiments are shown. Blue line indicates expression levels of DRs in parental Caco-2 cells. (TIF) [file pone.0021632.s012.tif]

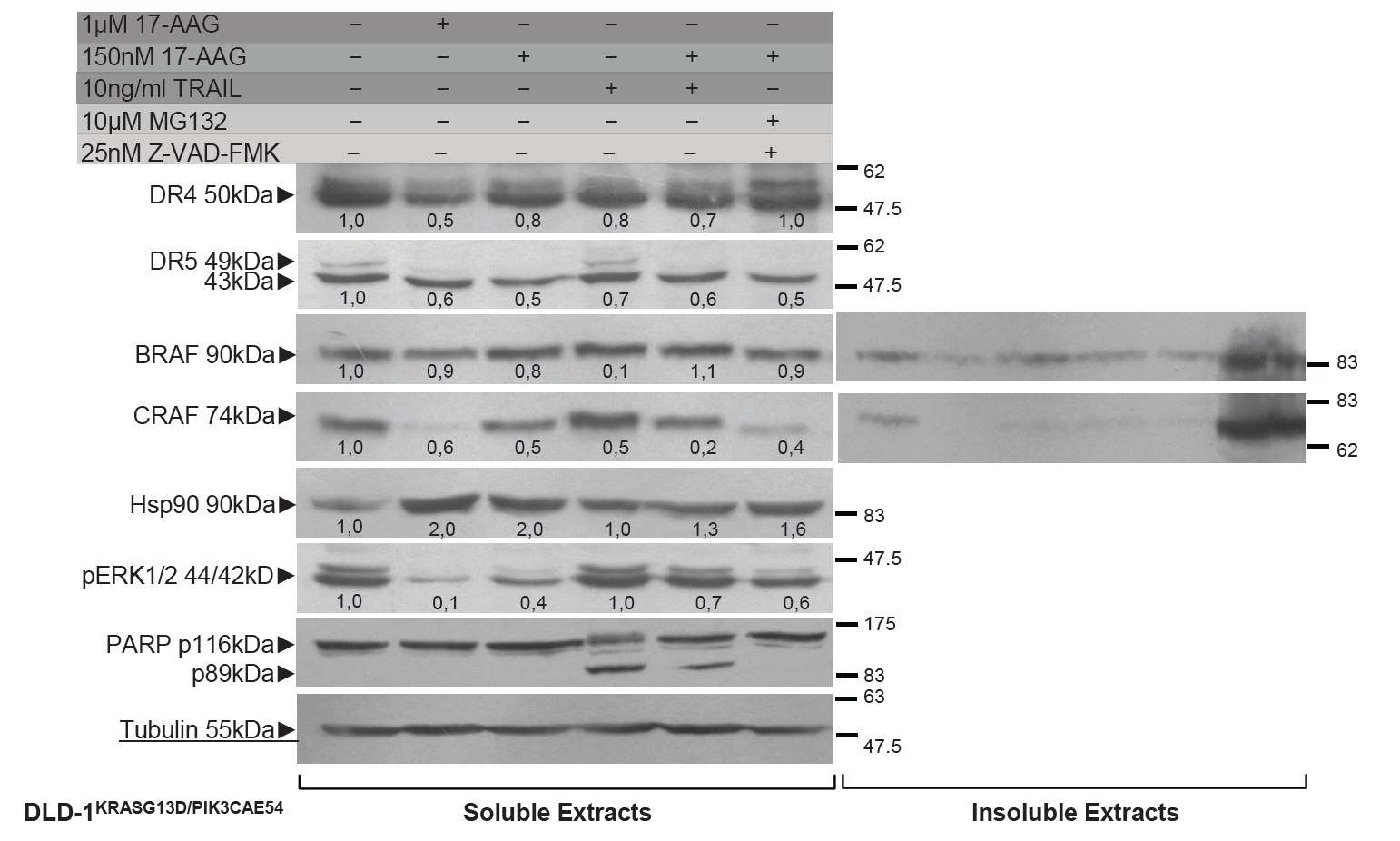

Supplement: Figure S13 — DR4 escapes 17-AAG dependent degradation in DLD-1 cells. Cells were left untreated or treated with 150 nM or 1 µM 17-AAG for 30 hours, or pre-treated with the 150 nM 17-AAG for 24 hours after which 10 ng/ml TRAIL was added for another 16 hours or 10 ng/ml TRAIL alone was added for 16 hours. Alternatively cells were pre-treated with 25 nM Z-VAD-FMK for 1 hour then 10 µM MG132 was added for another hour after which cell were treated with 150 nM 17-AAG for 24 hours. Protein extracts were separated into NP-40 soluble and NP-40 insoluble fractions and subjected to Western blot analysis. (TIF) [file pone.0021632.s013.tif]

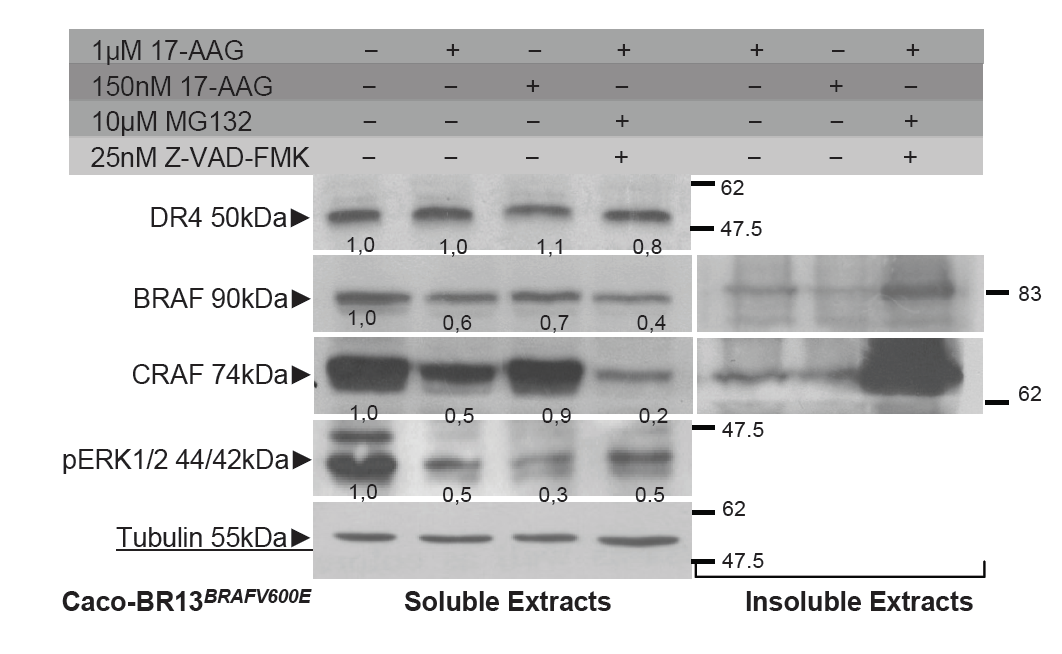

Supplement: Figure S14 — Caco-BR13 cells were left untreated or treated with a low 150 nM and a high 1 µM concentration of 17-AAG for 30 hours, or pre-treated with the 1 µM 17-AAG for 24 hours. Alternatively cells were pre-treated with 25 nM Z-VAD-FMK for 1 hour then 10 µM MG132 was added for another hour after which cell were treated with 1 µM 17-AAG for 24 hours. Protein extracts were subjected to Western blot analysis. Picture shown is representative of three independent experiments. (TIF) [file pone.0021632.s014.tif]

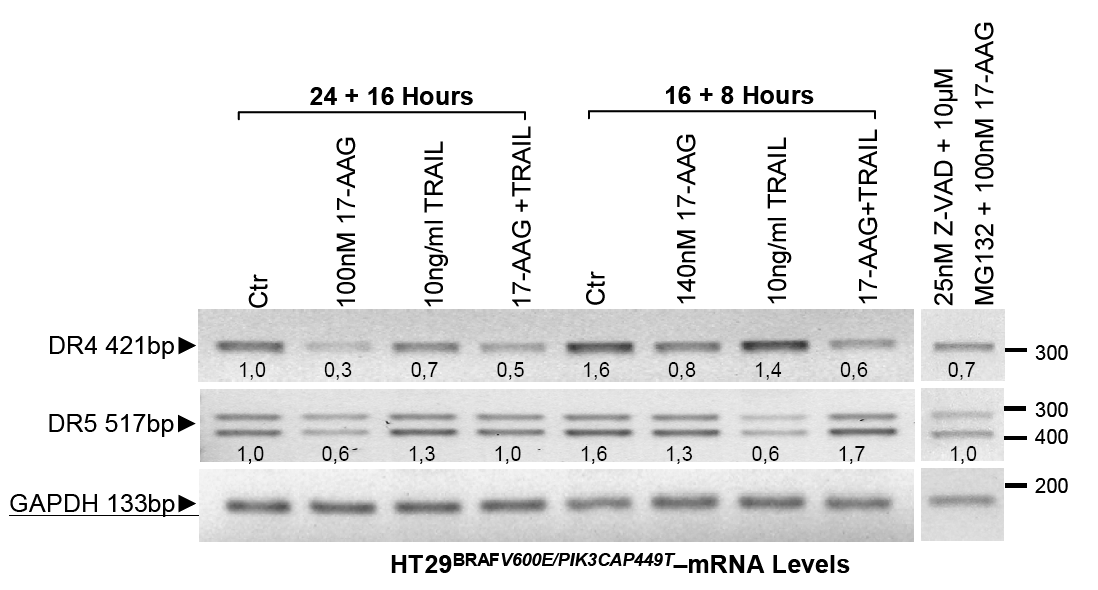

Supplement: Figure S15 — mRNA extracts from HT29 cells treated as indicated were analysis by RT-PCR with regard to TRAIL receptor, DR4 and DR5, transcriptional activity. (TIF) [file pone.0021632.s015.tif]
