# Supplementary material for: Selective BRAFV600E Inhibitor PLX4720, Requires TRAIL Assistance to Overcome Oncogenic PIK3CA Resistance
Source: PLoS One. 2011 Jun 27;6(6):e21632. doi: 10.1371/journal.pone.0021632 (PMC3124547; doi:10.1371/journal.pone.0021632)
Supplement: File S1 — Supplementary Materials and Methods . (DOC) [file pone.0021632.s016.doc]

**Supporting Experimental Procedures**

**Materials**

PLX4720 (918505-84-7) was purchased from Symansis (NZ Ltd, Timatu, New Zealand). Human recombinant SuperKiller cc-TRAIL (ALX-522-020), anti-DR4 (HS101) anti-DR5 (HS201) and FLIP (ALX-804-428) to be used for blocking experiments and immunoprecipitation analysis, 17-AAG (ALX-380-091), UO126 (ALX-270-237), PI-103 (ALX-270-460) and Z-VAD-FMK (ALX-260-138), were all purchased from Alexis (Biochemicals, Laussane, Switzerland). MG132 (Calbiochem, San Diego, CA). sulforhodamine B and Hoechst No. 33342 were purchased from Sigma-Aldrigh (Poole, UK). We used the following antibodies: B-Raf (sc-5284), pTyr^204^ERK (sc-7383), ERK2 (sc-1647), αTubulin (sc-8035), Cdc37 (sc-17758), cytochrome c (sc-13156), Bcl-2 (sc-7382), BID (sc-11423), Caspape-9 (sc-8355), BID (sc-11423), Ubiquitin (sc-8017) and cyclin D1 (sc-718) were purchased from Santa Cruz; pSer^445^B-Raf (2696), pSer^217/221^MEK1/2 (9121), pSer^473^ Akt (9271), Caspase-3 (9661), Caspase-6 (9762) and Caspae-8 (9746) were purchased from Cell Signaling (Danvers, MA, USA); C-Raf (610151) and FADD (F36620) were purchased from BD (Franklin Lakes, NJ, USA); pSer^338^Raf-1 (Upstate, 05-538); DR4 (1139) and DR5 (2019) for immunoblotting were purchased from ProSci (Poway, CA); DR4 (clone B5/B4; 199 DR4-02), DR5 (clone C11/B4; DR5-01-1) for immunofluorescence were purchased from Exbio (Vestec, Czech Republic); Hsp90 (SPA-830, Stressgen, Ann Arbor, USA) and Bax (MS-711-P, Thermo Scientific, Cheshire, UK); Alexa Fluor 555 goat anti-mouse and Alexa Fluor 488 goat anti-rabbit were purchased from Molecular Probes (Eugene, OR, USA). MitoTracker (M7512, Invitrogen, Oregon, USA).

**Cell Culture**

The Caco-2, Colo205, HT29, RKO, DLD-1 and SW620 human colon cell lines, and BRAF^V600E^ (Caco-BR) overexpressing clones and control (Caco-NEO9) empty vector clones derived from Caco-2 were cultured in Dulbecco's modified Eagle's medium supplemented with 10% fetal bovine serum (FBS), antibiotics and nonessential amino acids, all reagents were purchased from Invitrogen (Karlsruhe, Germany).

**Growth Inhibition Studies and Cytoxicity Assays**

We used the sulforhodamine B (SRB) assay as described previously ^1^ for growth inhibition and cytoxicity studies. Briefly, we seeded tumour cells into 96-well microtiter plates, allowed the cells to attach overnight, and then BRA^V600E^ selective inhibitor PLX4720 or Hsp90 inhibitor 17-AAG or apoptosis inducer TRAIL or PI3K inhibitor wortmanin alone in combinations were added to triplicate wells as indicated. Cells were exposed to drugs alone and in combinations as indicated. When required, cells were pre-incubated concomitantly with 60 μM MEK inhibitor UO126 or 10 μM proteasome inhibitor MG132, or 25 nM pan-caspase inhibitor Z-VAD-FMK for one hour prior to the main treatment. Thereafter, the cell number in treated versus control wells was estimated after treatment with 10% trichloroacetic acid and staining with 0.4% SRB in 1% acetic acid. The percentage of viable cell was plotted each time. SD was used for error bar generation. Differences were evaluated using Student's t test. P < 0.05 was considered significant. For blocking experiments cells were pre-incubated for 15 minutes with 2 μg/ml of the respective blocking antibody against DR4 and DR5 and then stimulated with TRAIL with and without pretreatment with 17-AAG. Photographs were taken using a Nikon Eclipse T-200 (Tokyo, Japan) inverted phase-contrast microscope equipped with an Olympus digital camera (Olympus SP-51OU2, Hamburg, Germany).

**ELISA Apoptotic Assay**

ELISA cell death kit by Roch (Roch, Indianapolis, IN) was alternatively used for the assessment and quantification of TRAIL-induced apoptosis according to the manufacturer’s protocols.

**Protein Immunobloting Analysis**

Cells were lysed in Nonidet P-40 (NP-40) buffer (50 mM Tris-HCl pH 7.5, 1% NP-40, 150 mM NaCl, 10% glycerol, 2 mM EDTA, 20 mM NaF, 10 mM PMSF, 2.5 mM Na3VO4 with 10 μM each leupeptine and aprotinin) and cleared by centrifugation. NP-40 insoluble fractions were lysed in 2% SDS sample buffer (Biolabs, New England, Beverly, MA) and boiled for 15 minutes. Protein concentrations were determined by using Bradford reagent (Bio-Rad, Hercules, CA, USA). Samples were separated in 10-12% SDS-PAGE, transferred to nitrocellulose, immunoblotted and detected by using the ECL detection system (Amersham Biosciences Uppsala, Sweden). Fold expression of all proteins analyzed (Supplementary Material and Methods) was determined after band intensity was established using Molecular Dynamics Image Quant Software (Amersham Biosciences, Uppsala, Sweden).

For the, immunoprecipitation studies cell lysates were precleared and equal amounts of protein (1,000 μg) were immunoprecipitated via primary overnight incubation with 6 μg BRAF antibody, 1.5 μg DR4 or normal anti-mouse IGg (negative control) at 4 ^o^C, followed by incubation with protein G-sepharose beads (GE Healthcare, Uppsala, Sweden) for 2 hours at 4 ^o^C. The immunoabsorbed pellets were washed three times with ice-cold wash buffer (0.05% Teween-20, 25 mM Tris pH 7.5, 150 mM NaCl, 10 mM MgCl_2_, 1 mM DTT) and resuspended in 2% SDS sample buffers. For Immunoprecipitation of the NP-40 insoluble fractions, pellets were resuspended in NP-40 lysis buffer and sonicated three times for 10 sec at 4˚C using an MSE Soniprep150 to give the insoluble fraction.

**DISC Immunoprecipitation by Streptavidin-Agarose Beads**

Sufficient number of cells was grown on a 10 cm tissue culture dishes per time point. Cells were pre-cooled for 15 minutes in the fridge. Next cells were incubate with 1 µg/ml biotinylated His-TRAIL first on ice for 15 minutes to saturate death receptors (DR) and then quickly warmed to 37 ^o^C for 30 minutes. A time 0’ control was included at this point to observe receptor status before DISC complex induction with Bio-TRAIL at 37 ^o^C. Cells were then transferred on ice and washed with ice-cold PBS. Cell lysis was performed in 200 µl of Lysis Buffer (20 mM Tris-HCl pH 7.5, 150 mM NaCl, 10% Glycerol, 10 mM EDTA, 1% NP-40, 10 mM NaF, 10 mM PMSF, 1 mM Na3VO4, with 10 μM each leupeptine and aprotinin) on ice for 30 minutes following centrifugation at 12,000 rpm for 30 minutes at 4 ^o^C. After centrifugation 20 µl of supernatant were collected in a separate tube for the ‘cell lysate’ control, and mixed with 10 µl of 2% SDS sample buffer. Remaining part of supernatant was mixed with 15 µl of equilibrated streptavidin-agarose beads (Pierce Biotechnology, Inc., Rockford, IL) according to the manufacturer and samples were rotated at 2000 rpm for 1 hour at 4 ^o^C. Streptavidin-agarose beads were then centrifuged at 2000 rpm for 2 minutes and supernatant was discarded after 20 µl of supernatant were collected in a separate tube for the ‘flow through’ control, and mixed with 10 µl of 2% SDS sample buffer. Beads were washed 5 times with the cell lysis buffer and protein complexes were eluted from the beads by the addition of 30 µl SDS sample buffer and heating at 95°C for 15 minutes. Proteins were separated in SDS-PAGE and immunoblotted for Caspase-8, FADD, DR4, DR5 and FLIP.

**Immunofluorescence Microscopy**

For immunostaining, cells were fixed with ice-cold methanol:acetone (8 : 1) or 4 % phosphate-buffer saline (PBS) - buffered paraformaldehyde (PFH). Cells were washed with PBS and quenched with 50 mM NH_4_Cl for 15 min in case of PFH. Nonspecific antibody binding was blocked with 5% FBS for 45 minutes at room temperature. The cells were incubated with indicated primary antibodies prepared in 1.5% FBS for 2 hours at room temperature, while the secondary antibody prepared in 1% FBS was applied to the cells for 1 hour also at room temperature. The nuclei were stained with Hoechst for 10 minutes and coverslips were mounted on glass slides in Gelvatol/ DABCO aqueous medium (Sigma-Aldrigh) and visualized with a Leica TCS SPE confocal laser scanning microscope (Leica Lasertechnik, Heidelberg, Germany). The objective lens used was 63x. The LAS AF software was used for image acquisition.

**Flow cytometry and apoptosis assays**

For immunostaining, 5x10^5^ cells were pre-incubated with blocking buffer (PBS with 0.2% gelatin, 0.1% sodium azide, and 20% human serum) on ice for 15 minutes and then incubated with 50 μg/ml anti-DR4 (DR-4-02, Exbio, Prague, Czech Republic) or anti-DR5 (DR5-01-1, Exbio) prepared in staining buffer (PBS containing 0.2% gelatin and 0.1% sodium azide) on ice for 30 minutes. After washing, cells were incubated with the secondary goat anti-mouse antibody coupled to phycoerythrin (IgG1-PE) (1:500), (1070–09, Southern Biotechnology Associates, Birmingham, Alabama) on ice for 30 minutes. After two final washes, the surface expression of the receptors on living cells (Hoechst negative) was analyzed using a LSRII flow cytometer (BD Biosciences). Annexin V (ALX-209-259, Alexis) - FITC/ Hoechst staining were used for the assessment and quantification of TRAIL-induced apoptosis according to the manufacturer’s protocols.
